# Genomic Clustering of differential DNA methylated regions (epimutations) associated with the epigenetic transgenerational inheritance of disease and phenotypic variation

**DOI:** 10.1186/s12864-016-2748-5

**Published:** 2016-06-01

**Authors:** M. Muksitul Haque, Eric E. Nilsson, Lawrence B. Holder, Michael K. Skinner

**Affiliations:** Center for Reproductive Biology, School of Biological Sciences, Washington State University, Pullman, WA 99164-4236 USA; School of Electrical Engineering and Computer Science, Washington State University, Pullman, WA 99164 USA

**Keywords:** DMR, Epigenetics, Control Region, Transgenerational, Computational Biology, Review

## Abstract

**Background:**

A variety of environmental factors have been shown to promote the epigenetic transgenerational inheritance of disease and phenotypic variation in numerous species. Exposure to environmental factors such as toxicants can promote epigenetic changes (epimutations) involving alterations in DNA methylation to produce specific differential DNA methylation regions (DMRs). The germline (e.g. sperm) transmission of epimutations is associated with epigenetic transgenerational inheritance phenomena. The current study was designed to determine the genomic locations of environmentally induced transgenerational DMRs and assess their potential clustering.

**Results:**

The exposure specific DMRs (epimutations) from a number of different studies were used. The clustering approach identified areas of the genome that have statistically significant over represented numbers of epimutations. The location of DMR clusters was compared to the gene clusters of differentially expressed genes found in tissues and cells associated with the transgenerational inheritance of disease. Such gene clusters, termed epigenetic control regions (ECRs), have been previously suggested to regulate gene expression in regions spanning up to 2-5 million bases. DMR clusters were often found to associate with inherent gene clusters within the genome.

**Conclusion:**

The current study used a number of epigenetic datasets from previous studies to identify novel DMR clusters across the genome. Observations suggest these clustered DMR within an ECR may be susceptible to epigenetic reprogramming and dramatically influence genome activity.

**Electronic supplementary material:**

The online version of this article (doi:10.1186/s12864-016-2748-5) contains supplementary material, which is available to authorized users.

## Background

An increasing number of studies in a wide variety of species have demonstrated that altered epigenetic information (such as DNA methylation, histone modifications, long non-coding RNA) can be passed on from one generation to the next through the germline (sperm and eggs) [1]. Environmental exposures such as nutrition, stress and toxicants can lead to germline epigenome changes (epimutations) that promote disease and phenotypic variation [1, 2]. Exposure induced epimutations include specific changes in the pattern of DNA methylation, which are known as differential DNA methylation regions (DMRs). Environmentally induced epigenetic transgenerational inheritance promotes changes in gene expression in tissues and cells of individuals unexposed transgenerational descendants of ancestors exposed to environmental insults. Previous analysis of transgenerational differential DNA methylation regions (DMRs) in sperm suggested the existence of exposure-specific DMRs [3]. The current study was designed to investigate the genomic associations of these transgenerational epimutations. Previous studies have also identified cell and tissue specific transgenerational alterations in gene expression (transcriptomes) following ancestral toxicant exposure [4–6]. The differentially expressed genes in a transgenerationally altered transcriptome have previously been shown to often cluster on the genome [4]. The associations of transgenerationally inherited epimutations and these transgenerational transcriptome changes are investigated in the current study. The identification of DMR clusters and their relation to changes in gene expression provides insight into epigenetic mechanisms that regulate gene expression in chromosomal regions.

Genomic clusters are defined as statistically significant over represented molecular features in a region of the genome [4]. The clustering of molecular features on the genome has been proposed to provide functionally important associations of genetic and epigenetic phenomena [7, 8]. For example regions which contain a cluster of genes might have a coordinated regulation of gene expression [4]. Similarly, the epigenome also has certain regions which exhibit groupings of epigenetic features or associated genomic components [9, 10].

Gene clusters are natural phenomena that have been identified and reviewed [7]. There are two different types of gene clusters to consider. In the first type genes can be clustered together based on the similarity of their gene functions [11]. In that case a cluster can define a gene family, such as that seen with the developmentally important *Hox* genes [12, 13]. Therefore, gene clusters can encode functionally related genes and proteins to allow for an efficient regulation of gene expression. These clustered genes can reside on the same chromosome or on different chromosomes [14]. A second type of gene clustering can be defined by genes that are clustered based on their genomic location or proximity to each other. Such gene clusters always start and end on the same chromosome. These clustered genes are often within a few million base pairs distance of each other. Gene clusters are thought to be due in part to evolutionary and functional relationships among the genes [15]. The clustering of genes has been shown to have an important impact on biological processes. The relationship of genomic clusters associated with transgenerational differentially expressed gene clusters and differential DNA methylation regions (DMRs) clusters are investigated in the current study.

Previous studies have investigated gene clustering [7, 8]. For example, clustering of human transcriptome data was performed to find links between transcriptome regulation and chromosomal gene order [16]. Groups of genes in clusters which are regulated by the same transcription factors have been identified [16]. Another study used genome contexts to remove noise and identify clusters of functionally related genes [17]. Clusters as large as 118 genes were found to be common in three different species’ genomes [18]. Another study examined 25 clusters of genes which appear to be regulated by the chromatin remodeling complex TRX (the trithorax group). This was done with genome-wide expression studies of the trx mutant in the Drosophila genome [8]. Several studies have examined clustering of specific gene families [19, 20]. These observations on gene clusters have been extended in a recent analysis of DNA methylation data. A novel clustering approach called adjacent site clustering (A-clustering) detects neighboring CpG sites that are correlated with methylation changes [21].

Previous studies by our laboratory applied a statistical clustering method to transgenerational datasets of altered gene expression from female and male tissues [4], and from purified cell types including Sertoli cells [5], granulosa cells [6], and primordial germ cells (PGC) [22]. The cell specific transcriptome data was based on micro-array studies that measured mRNA expression from different tissues from both male and female transgenerational F3 generation vinclozolin versus control lineage rats [5, 6, 22]. The Sertoli cell and granulosa cell transgenerational transcriptome datasets from adult F3 generation vinclozolin versus control lineage somatic cells are associated with the onset of testis and ovarian disease, respectively [5, 6]. Examination of each tissue’s transgenerational transcriptome identified tissue specific alterations in those transcriptomes [4]. Using data from these analyses and running them through a clustering analysis produced a number of clusters of differentially expressed genes [4]. A sliding window based clustering technique was used to find groups of differentially expressed gene sites based on their distance from each other [4]. Since there is a natural gene clustering background due to the pre-existing clustering of genes on chromosomes, those clusters computed from all the genes in the genome were considered in identification of internal background gene clusters. In addition to cell and tissue specific transgenerational differential gene expression clusters, global differentially expressed gene clusters were identified by combining the chromosomal location data from all the tissues and cell types [4]. The clusters from the transgenerational transcriptome data suggested a regional regulation of gene expression in those cluster areas that were termed epigenetic control regions (ECR) [4]. It is hypothesized that within an ECR many genes are epigenetically up-regulated or down-regulated in concert. Those genes that would normally be expressed in any specific tissue would be subject to that ECR’s regional up- or down-regulation. The investigation of genomic clusters will help to identify potential regulatory regions in the genome which can use epigenetic mechanisms to regulate gene expression across millions of bases on a chromosome region. The current study extends these previous studies that used transgenerational transcriptome cluster analysis to now investigate the clustering of the associated sperm DMR epimutations (i.e. DMR clusters) and relationships with differential gene expression clusters (i.e. gene clusters).

## Results

### Gene expression clusters

A genome cluster analysis procedure was developed that involved a 50,000 bp sliding window analysis of the genome to identify regions that have statistically over-represented numbers of genes and genomic features [4], as described in the Methods. The computer R-code used is a language for statistical computing from the R Foundation for Statistical Computing, Vienna, Austria. (ISBN 3-900051-07-0, URL http://www.R-project.org). The R-code developed for the current study is presented in Additional file 1: Figure S1. Initially the transgenerational differentially expressed gene clusters were identified as previously described [4]. The different types of transgenerational gene expression data and their cluster information are presented in Table 1A for F3 generation exposure lineages. For the transgenerational female tissue array (FT) (involving ovary, uterus, heart, kidney and liver) RNA expression data there were 1052 genes which were differentially expressed. These genes clustered into 28 groups having a total of 221 genes (21 % of total sites) in these clusters. The male transgenerational tissues (MT) (involving testis, prostate, heart, kidney, liver) had 2763 differentially expressed genes that involved 40 clusters with 727 genes (26 % of total sites). Sertoli cells and Granulosa cells have 24 and 22 gene clusters respectively from 430 and 479 genes. The primordial germ cells have 15 gene clusters from 138 genes. Changes in gene numbers in the current study and the original study [4] was due to not using the seminal vesicle in the male tissues and reducing the number of redundant overlapped genes.

**Table 1:** Gene and DMR cluster overlap

(A) Gene clusters from FT, MT, SC, GC, PGC data sets and DMR cluster data sets							
	Number of Sites	Total Clusters	Gene in Cluster	Data Type			
Female Tissue (FT)	1052	28	221	Differential Gene Expression			
Male Tissue (MT)	2763	41	727	Differential Gene Expression			
Sertoli Cell (SC)	430	24	101	Differential Gene Expression			
Granulosa Cell (GC)	479	22	111	Differential Gene Expression			
Primordial Germ Cell (PGC)	739	15	138	Differential Gene Expression			
DMR	776	21	194	Differential DNA Methylation			
Background (BKG)	20917	59	7132	Locations of All Genes			
(B) Overlap of gene and DMR clusters between FT, MT, SC, GC, PGC, and DMR and background (BKG)							
	FT(28)	MT(41)	SC(24)	GC(22)	PGC(15)	DMR(21)	BKG(59)
FT(28)	28	14	8	8	2	5	18
MT(41)	14	41	13	14	6	11	31
SC(24)	8	13	24	4	4	2	15
GC(22)	8	14	4	22	5	4	17
PGC(15)	2	6	4	5	15	2	14
DMR(21)	5	11	2	4	2	21	15
BKG(59)	18	31	15	17	14	15	59
(C) Clusters overlap among datasets after removal of background gene clusters							
	FT(12)	MT(11)	SC(9)	GC(5)	PGC(2)	DMR(7)	
FT(12)	12	1	0	0	0	0	
MT(11)	1	11	0	1	1	1	
SC(9)	0	0	9	0	0	0	
GC(5)	0	1	0	5	0	0	
PGC(2)	0	1	0	0	2	0	
DMR(7)	0	1	0	0	0	7	

(A) Gene clusters from FT, MT, SC, GC, PGC, BKG, as well as DMR clusters. The total number of clusters in each of these datasets, and total number of genes or DMR in these clusters. (B) The overlap in number of sites among these clusters. (C) The unique clustering for each dataset after removal of the gene cluster background

A gene cluster analysis also examined all the gene locations in the entire genome to find the internal background (BKG) gene clustering pattern. This information was obtained from the Affymetrix annotated gene set and was referred to as (BKG) clusters. From all the 20,917 genes examined in the rat genome 59 BKG clusters were identified. A total of 7132 genes were part of these 59 BKG clusters. These background gene clusters are anticipated to be similar in nature to other gene clusters in the genome, but were separated to clarify that additional gene clusters were identified independent of these internal background gene clusters. When each of the transgenerational transcriptome datasets (a total of 6544 sites) was separately clustered they formed into 154 clusters. The 59 background gene clusters were overlapped with these 154 gene clusters. Any of the background gene clusters which overlapped with the 154 ECR clusters were removed which left 45 transgenerational gene clusters for all of the tissue types.

**Fig. 1:**
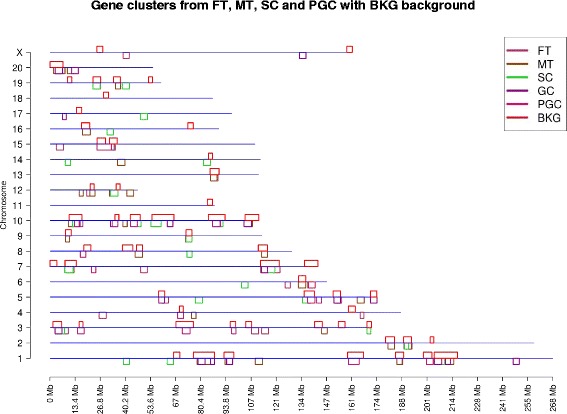
Gene clusters with background. Chromosome locations of gene clusters from FT (Female Tissue), MT (Male Tissue), SC (Sertoli Cell), GC (Granulosa Cell), PGC (Primordial Germ Cell) compared with BKG cluster background. The chromosome number and size (Mb) are shown and the different gene clusters are color coded below line with the background BKG gene cluster above the line

A large number of clusters overlap between the FT, MT, SC, GC, PGC and BKG as can be seen from Fig. 1 and Table 1B. The total number of gene clusters varies from 15 to 28, while the number of genes in each cluster varies from 111 to 7132. Detailed information about the different gene expression datasets including gene cluster locations and regulated genes within each cluster are presented in Additional file 2: Table S1 (Male Tissue), Additional file 3: Table S2 (Female Tissue), Additional file 4: Table S3 (Sertoli Cell), Additional file 5: Table S4 (Granulosa Cell), and Additional file 6: Table S5 (Primordial Germ Cell). The associated gene classification categories are also presented to clarify potential functional impacts.

**Fig. 2:**
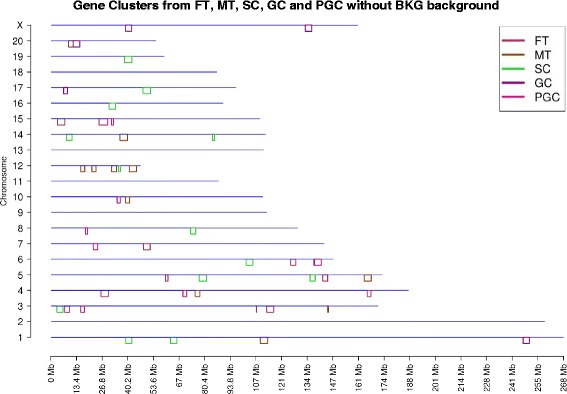
Gene clusters without background. Chromosome location of gene clusters from FT, MT, SC, GC, PGC without BKG cluster background. The chromosome number and size (Mb) are shown with color coded gene clusters below line

Since there is a background signal due to the inherent clustering of genes in the genome, the BKG gene cluster information was extracted and removed from the original clusters. After the gene background information was removed (as described in the Methods section), the number of unique clusters reduced in the female gene set to 12 and the number of unique clusters reduced in the male tissue gene set to 11. These clusters only had one area of overlap between the MT and FT comparison, and higher overlap when the background was considered. Similarly, unique clusters are shown from Sertoli cell (SC) (9 clusters), granulosa cell (GC) (5 clusters), and PGC (2 clusters). This information is shown in Fig. 2 and Table 1C. Comparing Table 1B with Table 1C it can be seen after removing the genome cluster BKG information that the number of unique clusters for each of the gene datasets and the overlap among them decreased. As stated, the initial gene clusters in the background are similar in nature functionally to those not distinct from the background. However, the presence of additional gene clusters not in the background suggests new clusters appear in the transgenerational transcriptomes. Gene clusters, both with and without BKG subtraction, were correlated with the DMR clusters.

### Differential DNA Methylation Region (DMR) clusters

**Fig. 3:**
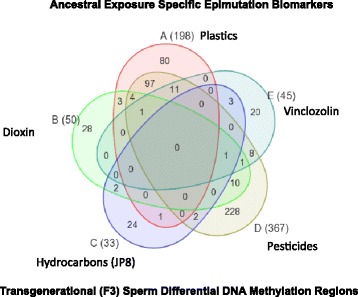
Exposure specific differential DNA methylation regions (DMR) data set overlap. The promoter associated DMR are listed for each data set (number in brackets). The Venn diagram identifies numbers of overlaps and those unique to the various exposures (plastics, vinclozolin, pesticides, hydrocarbons and dioxin). Modified from [3]

**Fig. 4:**
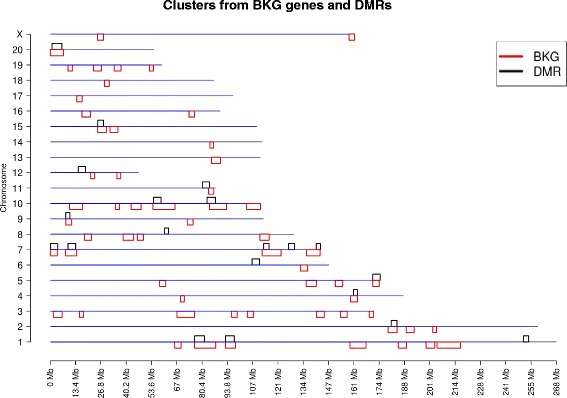
Chromosome location of BKG gene clusters versus DMR clusters. The chromosome number and size (Mb) are presented with the DMR in black above the line and BKG in red below the line

**Fig. 5:**
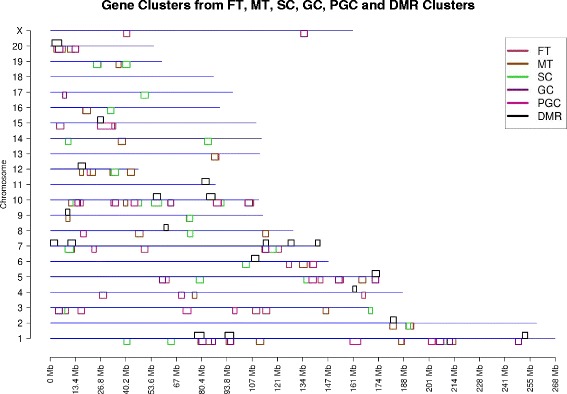
Chromosome location of gene clusters and DMR clusters. Chromosome location of gene clusters from FT, MT, SC, GC, PGC compared to DMR clusters. The chromosome number and size (Mb) are presented with DMR cluster in black above the line and gene clusters color coded below the line

**Fig. 6:**
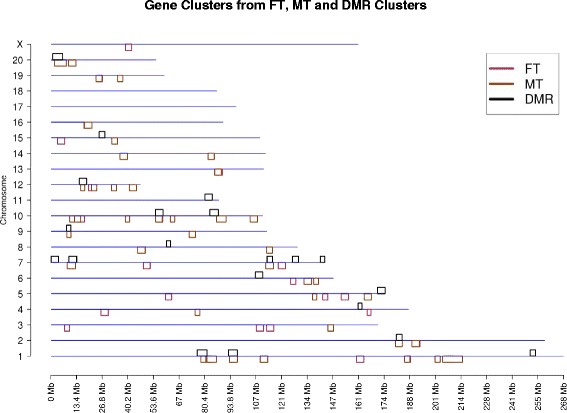
Male and female gene clusters versus DMR clusters. Chromosomal locations of gene clusters from FT, MT compared to DMR clusters. The chromosome number and size (Mb) are presented with DMR clusters in black above the line and gene clusters color coded below the line

The transgenerational sperm DMR data were collected from previous studies [3]. These studies identified chromosomal locations of transgenerational DMR in the F3 generation sperm following F0 generation exposure to environmental toxicants including dioxin [23], plastics [24], pesticides [25], jet fuel [26], bisphenol A [24] and vinclozolin [27]. Interestingly, no DMR overlapped between all the data sets and the majority of DMR were exposure specific [3], Fig. 3. The combined transgenerational DMR from these previous studies total 776 DMR sites that clustered into 21 DMR clusters. The clusters contained 194 of the DMRs (25 % of the entire DMRs from these studies). The DMR clusters are shown in Fig. 4. One DMR cluster was present in a highly repetitive uncharacterized chromosomal region and so could not be assigned to a specific chromosome. DMR clusters exhibited some overlap with BKG gene clusters, as would be expected since the DMR data was collected using gene promoter microarrays. These DMR clusters also overlapped with gene clusters from FT, MT, SC, GC and PGC (Fig. 5). A subset of these data show the 21 clusters from the DMR are overlapped with the 40 male and the 28 female tissue transgenerational gene clusters (Fig. 6). There are 11 intersections of the male tissue array clusters with the DMR clusters, and 5 intersections of the female tissue array clusters with the DMR clusters. Therefore, many other DMR clusters overlapped with the gene clusters.

Detailed information on each of the different gene clusters and DMR clusters are presented in Table 1. The final row shows information of the internal background gene clusters, which were calculated using all the available genes in the genome. The detailed information for each of the gene clusters in the male and female tissue array data sets are presented in Additional file 2: Tables S1 and Additional file 3: Table S2, respectively. The detailed information for the DMR clusters are presented in Additional file 4: Table S3 for Sertoli cell, Additional file 5: Table S4 for granulosa cells, Additional file 6: Table S5 for primordial germ cells, and Additional file 7: Table S6 for the sperm. Each supplemental table shows the number of clusters, the total number of sites (genes or DMR) creating each of the clusters, and their locations on each chromosome. For all clusters the statistical *p*-value reflecting the over representation of genes or DMR in the cluster is presented.

**Fig. 7:**
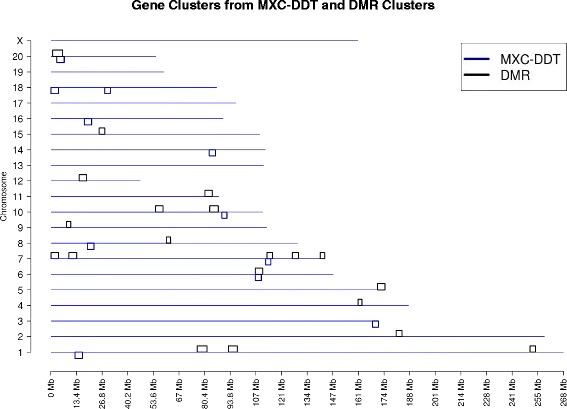
Normal MXC-DDT DMR cluster versus genomic DMR clusters. Chromosome locations of DMR clusters versus MXC and DDT DMR. The chromosome number and size (Mb) are presented with DMR clusters in black above the line and MXC-DDT DMR clusters below the line

The sperm DMR clusters have been created from 5 different toxicant induced transgenerational F3 generation lineages sperm (ancestral dioxin, plastics, jet fuel, bisphenol A or vinclozolin) and the somatic cell DMR clusters (Sertoli cell, granulosa cell and primordial germ cell) were derived from the vinclozolin induced F3 generation lineages. Since the DMR clusters were derived from these known data sets, an independent test was done to investigate the overlap with DMR clusters which are formed of DMR sites from additional treatments. As can be seen from Fig. 7, the identified DMR clusters also overlap with the 11 clusters from the combined methoxychlor (MXC) [28] and dichlorodiphenyltrichloroethane (DDT) [29] DMR datasets. These two datasets are used as positive DMR sets for validation purposes. Observations identify 3 overlaps in chromosome 6, 7 and 20 with the MXC and DDT DMR and the DMR clusters identified, Fig. 7. This validation helps suggest that the clustering approach and analysis are finding chromosomal regions where epigenetic remodeling (DNA methylation) occurs.

**Fig. 8:**
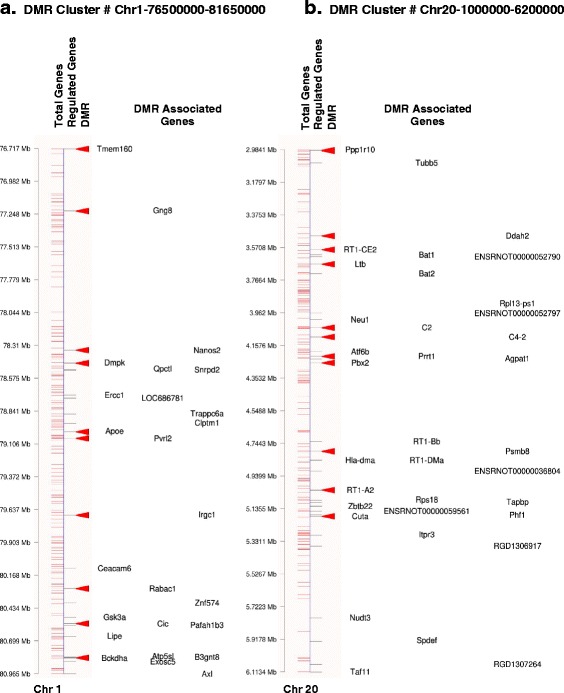
Representative DMR clusters. **a** DMR cluster on chromosome 1 (76500000-81650000) **b** DMR cluster on chromosome 20 (2900000-6200000). The chromosome map with megabase location is indicated for all genes, regulated genes and DMR (red arrowhead). Gene symbols associated with regulated genes and DMR associated genes are listed

Representative examples of two DMR clusters with overlap with a gene cluster is shown in Fig. 8. Observations show a detailed view of associated gene clusters with the DMR cluster. Representative DMR clusters were chosen to demonstrate cluster size, the number of differentially expressed gene sites that make up the epigenetic control region (ECR) within the gene cluster, and the position of DMR within the cluster.

## Discussion

The primary objective of the current study was to identify potential DMR clusters and see if they correlate with gene clusters in the genome. The DMR were previously identified in promoter locations of genes and appear to be potential regulatory sites. A variety of different environmental toxicant exposures (dioxin [23], plastics [24], pesticides [25], jet fuel [26] and vinclozolin [27]) were separately found to promote the epigenetic transgenerational inheritance of disease involving differential DNA methylation regions (DMR) in F3 generation male sperm. These F3 generation sperm DMR previously identified were used as the sperm DMR for the current study. The F3 generation vinclozolin lineage animals also had Sertoli cells, granulosa cells and primordial germ cells isolated and DMR identified which were used in the current study as somatic cell DMR datasets, as previously described [4]. These DMR datasets were used for the DMR cluster analysis. In addition, tissue specific differential gene expression in the F3 generation vinclozolin lineage tissues and somatic cells were found to cluster and these regions have been called epigenetic control regions (ECRs) [4]. These gene clusters previously identified were used in the current study to compare with the DMR clusters identified. The hypothesis examined was that within these chromosomal segments of the epigenetic control regions that epigenetic regulatory sites exist, such as DMRs and non-coding RNAs, that regulate gene expression within the ECR. One purpose of the current study was to examine whether DMRs, or clusters of DMRs, were found in the previously identified ECR defined by clusters of differentially expressed genes. DMR clusters identified were found to overlap with the gene expression clusters (Figs. 5 and 6), although this overlap is less if background gene clustering is subtracted from total gene clusters (Table 1C and Fig. 4). Observations identified the existence of DMR clusters that overlap with gene clusters. Therefore, the ECR will likely involve the statistical over representation of genes in a 2–5 megabase region which can also involve clustering of DMR to influence the gene expression in these regions. Since the data obtained only used promoter associated DMR, a genome-wide analysis of DMR sites may in the future reveal a greater overlap with gene clusters and ECR. This is a limitation of the current study that needs to be considered as distal gene regulation has been previously observed in relationship to DMR and long non-coding RNA [30].

A previous study examined clustering of CpG sites within DMR regions. Sofer et al. (2013) examined 460,337 CpG sites and constructed CpG clusters. In the first analysis they used a Spearman correlation-based distance measure using average distance type and a distance threshold of D = 0.2, such that to merge two clusters all the sites in these two clusters should be within a threshold distance of D. This produced 7741 CpG clusters with at least 2 CpG sites and with the largest having 52 sites. They then used an exposure effect (i.e. treatment) cutoff of 0.02 size and finally got 402 sites with differential methylation (DMR) involving a minimum of 2 CpG and a maximum of 3 CpG sites. For the second analysis using a less stringent criteria of the distance (a single distance type indicates a cluster having only a single site) with D = 0.5 they found 17515 CpG clusters with at least 2 sites and with the largest cluster having 59 sites. Similar to the first experiment they used an exposure effect size of > 0.02 and found 641 DMR [21]. In this study initially the clustered methylated DNA sites were identified and then afterwards checked for the effect of experimental treatments on the clusters. In the current approach the clusters are constructed directly from previous identified regulated DMR sites. Although this study does not directly correlate to the current study, the concept that clusters of regulated DMR may exist is suggested in both cases.

Previously a number of transgenerational differential gene expression clusters were identified and termed epigenetic control regions (ECR) [4]. As described in the current study gene clusters were identified separately and from a variety of tissues and cell types. In the current study, the contribution of inherent background gene clustering was investigated. The clustering of genes within the genome has been previously observed. Although the nature of inherent gene clustering and induced gene clustering is anticipated to be the same, the contribution of the background gene clusters was examined. All the genes from the rat genome (collected from Affymatrix gene annotation files) were used to find the underlying background (BKG) gene cluster pattern on all the chromosomes in the genome. Using 20,917 genes from the gene annotation files a total of 59 clusters were found. Approximately one third of the known genes (7,132) were in these 59 clusters. This information was used as background gene cluster information. These background ECR are thought to be potential ECR, but the relative contribution was assessed compared to the transgenerational gene clusters examined.

The transgenerational transcriptome dataset consisting of 6544 gene sites was clustered to form 154 gene clusters. The background gene clusters were removed from these and resulted in 45 distinct gene clusters. These 45 gene clusters of transgenerational differentially expressed genes, which are not associated with background gene clustering, indicates that novel regions of the genome can be affected by ancestral exposure to toxicants, as well as the background gene clusters. Epigenetic transgenerational inheritance of differential gene expression in part involves gene clusters in specific chromosomal regions. This supports the previous reported concept that these sites are epigenetic control regions (ECR). The overlapped background gene clusters were removed to determine if there are gene clusters forming for each of the environmental exposures. Since each of the toxicants had their own cluster after removal of background gene clusters, observations indicate that each tissue type had their own unique ECR signature. There were 7 unique clusters remaining for each of the expression datasets. A combination of the differentiated gene clusters that aligned with background gene clusters and the 45 non-background gene clusters are speculated to be epigenetic control regions and important for the epigenetic regulation of genome activity.

Transgenerational sperm DMR sites were used from previous experiments and in a cluster approach similar to the gene clusters associated with ECR sites [4]. A total of 776 DMR sites were used as input to the clustering analysis (Additional file 1: Figure S1) and 21 DMR clusters were identified. These 21 DMR clusters were overlapped with the ECR clusters (Figs. 5 and 6). A noticeable amount of overlap was found with all gene clusters as well (Table 1B). This study was done to check if gene clusters associated with ECR overlap with DMR clusters. DNA methylation may be part of the epigenetic mechanism that regulates gene expression within the ECR region. The effect of the inherent background gene clusters in the genome on interpretation of gene clusters and DMR clusters was considered. The concern was that the existence of background gene clusters alter the interpretation of the gene clusters identified. Therefore, the locations of gene expression for the gene clusters and identification of the DMR clusters were presented both with and without subtraction of background gene clusters. Although unique gene clusters and DMR clusters were identified without the background gene clusters, the inherent background gene clusters are expected to have the same characteristics and function as general ECR. The speculation is made that evolutionary forces that promote clustering of genes on the genome involve epigenetic factors such as DNA methylation that contribute to regional gene regulation. Therefore, it is anticipated that a functional importance exists for the co-localized gene expression within a gene cluster and the co-localized DMR clusters have a role in the gene regulation in the ECR. The causal relationship of these gene and DMR clusters involving epigenetic regulation needs to be further established. Future studies involving genome wide analysis of DMR and an analysis of a wider variety of tissues and cell types for gene expression will facilitate this analysis in the future.

## Conclusions

The current study demonstrates that environmentally induced epigenetic transgenerational inheritance of F3 generation sperm DMRs often cluster on the genome and associate with the previously identified gene clusters within epigenetic control regions (ECR). Similar observations were made with transgenerational somatic cell DMR clusters and gene clusters. Therefore, within the ECR the gene cluster is speculated to be regulated by the associated DMR cluster. The causal link between the gene cluster and DMR cluster remains to be established, but the potential presence of ECR that are regulated by DMR clusters provide a novel mechanism for the control of genome activity. The ability of a variety of different environmental exposures to promote the epigenetic transgenerational inheritance of sperm DMR clusters and tissue specific gene expression profiles is anticipated to utilize in part such a regulatory mechanism.

## Methods

### Previous animals procedures

All experimental protocols involving rats were pre-approved by the Washington State University Animal Care and Use Committee. Hsd:Sprague Dawley®™SD®™ female and male rats of an outbred strain (Harlan) were maintained in ventilated (up to 50 air exchanges/hour) isolator cages containing Aspen Sani chips (pinewood shavings from Harlan) as bedding, on a 14 h light: 10 h dark regimen, at a temperature of 70 F and humidity of 25 to 35 %. Rats were fed ad libitum with standard rat diet (8640 Teklad 22/5 Rodent Diet; Harlan) and ad libitum tap water for drinking.

At proestrus as determined by daily vaginal smears, the female rats (90 days of age) were pair-mated with male rats (120 days). On the next day, the pairs were separated and vaginal smears were examined microscopically. In the event sperm were detected (day 0) the rats were tentatively considered pregnant. Vaginal smears were continued for monitoring diestrus status in these rats until day 7. Pregnant rats were then given daily intraperitoneal injections of vinclozolin or other toxicants with an equal volume of sesame oil (Sigma) on days E-8 through E-14 of gestation [31]. Treatment groups were Control (DMSO vehicle) and exposure. The pregnant female rats treated with DMSO or vinclozolin were designated as the F0 generation.

The offspring of the F0 generation were the F1 generation. The F1 generation offspring were bred to other F1 animals of the same treatment group to generate an F2 generation and then F2 generation animals bred similarly to generate the F3 generation animals. No sibling or cousin breedings were performed so as to avoid inbreeding. Note that only the original F0 generation pregnant females were injected with the DMSO or vinclozolin.

Six female and six male rats of F3 generation Control and Vinclozolin lineage at 120 days of age were euthanized by CO2 inhalation and cervical dislocation. Tissues including testis, prostate, seminal vesicle, kidney, liver, heart, ovary and uterus were dissected from rats and were processed and stored in TRIZOL (Invitrogen) at -80 °C until RNA extraction. High quality RNA samples were assessed with gel electrophoresis and required a minimum OD260/280 ratio of 1.8. Three samples each of control and exposure F3 generation lineage tissues were applied to microarrays. For each of three Vinclozolin or Control microarray sample, RNA from two rats were pooled. The same pair of rats were used for each tissue type.

### Previous microarray analysis

The microarray hybridization and scanning was performed by the Genomics Core Laboratory, Center for Reproductive Biology, Washington State University, Pullman, WA using standard Affymetrix reagents and protocol. Briefly, mRNA was transcribed into cDNA with random primers, cRNA was transcribed, and single-stranded sense DNA was synthesized which was fragmented and labeled with biotin. Biotin-labeled ssDNA was then hybridized to the Rat Gene 1.0 ST microarrays containing more than 30,000 transcripts (Affymetrix, Santa Clara, CA, USA). Hybridized chips were scanned on Affymetrix Scanner 3000. CEL files containing raw data were then pre-processed and analyzed with Partek Genomic Suite 6.5 software (Partek Incorporated, St. Louis, MO) using an RMA, GC-content adjusted algorithm. Raw data pre-processing was performed in 11 groups, one for each male or female tissue, as well as the Sertoli cell, granulosa cell and primordial germ cells. Comparison of array sample histogram graphs for each group showed that data for all chips were similar and appropriate for further analysis.

The microarray quantitative data involves signals from an average 28 different oligonucleotides (probes) arrayed for each transcript and many genes are represented on the chip by several transcripts. The hybridization to each probe must be consistent to allow a statistically significant quantitative measure of resulting gene expression signal. In contrast, a quantitative PCR procedure uses only two oligonucleotides and primer bias is a major factor in this type of analysis. Therefore, we did not attempt to use PCR based approaches as we feel the microarray analysis is more accurate and reproducible without primer bias.

All microarray CEL files from this study have been deposited with the NCBI gene expression and hybridization array data repository GEO (GEO series accession number: GSE35839) and can be also accessed through www.skinner.wsu.edu. For gene annotation, Affymetrix annotation file RaGene1_0stv1.na32.rn4.transcript.csv was used.

### Previous sperm DNA isolation and methylated DNA immunoprecipitation (MeDIP)

Sperm heads were separated from tails through sonication following previously described protocol (without protease inhibitors) [32] and then purified using a series of washes and centrifugations [33] from a total of nine F3 generation rats per treatment lineage that were 120 days of age. DNA extraction on the purified sperm heads was performed as previously described [27]. Equal concentrations of DNA from individual sperm samples were then used to produce pools of DNA material. Three DNA pools were produced in total per treatment, which contained the same amount of sperm DNA from three animals. Therefore a total of 45 animals were used for building three DNA pools per treatment for the 4 experimental groups plus controls. These DNA pools were then used for methylated DNA immunoprecipitation (MeDIP). MeDIP was performed as follows: 6 μg of genomic DNA was subjected to series of three 20 pulse sonications at 20 % amplitude and the appropriate fragment size (200-1000 ng) was verified through 2 % agarose gels; the sonicated genomic DNA was resuspended in 350 ul TE and denaturated for 10 min at 95 °C and then immediately placed on ice for 5 min; 100 ul of 5X IP buffer (50 mM Na-phosphate pH7, 700 mM NaCl, 0.25 % Triton X-100) was added to the sonicated and denatured DNA. An overnight incubation of the DNA was performed with 5 ug of antibody anti-5-methylCytidine monoclonal from Diagenode S.A (Denville, NJ) at 4 °C on a rotating platform. Protein A/G beads from Santa Cruz (Santa Cruz, CA) were prewashed on PBS-BSA 0.1 % and resuspended in 40 ul 1X IP buffer. Beads were then added to the DNA-antibody complex and incubated 2 h at 4 °C on a rotating platform. Beads bound to DNA-antibody complex were washed 3 times with 1 ml 1X IP buffer; washes included incubation for 5 min at 4 °C on a rotating platform and then centrifugation at 6000 rpm for 2 min. Beads-DNA-antibody complex were then resuspended in 250 ul digestion buffer (50 mM Tris HCl pH 8, 10 mM EDTA, 0.5 % SDS) and 3.5 ul of proteinase K (20 mg/ml) was added to each sample and then incubated overnight at 55 °C on a rotating platform. DNA purification was performed first with phenol and then with chloroform:isoamyl alcohol. Two washes were then performed with 70 % ethanol, 1 M NaCl and glycogen. MeDIP selected DNA was then resuspended in 30 ul TE buffer.

### Previous tiling array MeDIP-Chip analysis

Roche Nimblegen’s Rat DNA Methylation 3x720K CpG Island Plus RefSeq Promoter Array was used, which contains three identical sub-arrays, with 720,000 probes per sub-array, scanning a total of 15,287 promoters (3,880 bp upstream and 970 bp downstream from transcription start site). Probe sizes range from 50–75 mer in length with the median probe spacing of 100 bp. Three different comparative (MeDIP vs MeDIP) hybridizations experiments were performed for each experimental group versus control, each encompassing DNA samples from 6 animals (3 treatment and 3 control groups) and 3 sub-arrays. MeDIP DNA samples from experimental groups were labeled with Cy3 and MeDIP DNA samples from the control group were labeled with Cy5.

### Previous bioinformatic and statistic analyses of chip data

For each comparative hybridization experiment, raw data from both the Cy3 and Cy5 channels were imported into R (R Development Core Team (2010), R: A language for statistical computing, R Foundation for Statistical Computing, Vienna, Austria. ISBN 3-900051-07-0, URL http://www.R-project.org), checked for quality and converted to MA values (M = Cy5-Cy3; A = (Cy5 + Cy3)/2). The following normalization procedure was conducted. Within each array, probes were separated into groups by GC content and each group was separately normalized, between Cy3 and Cy5 using the loess normalization procedure. This allowed for GC groups to receive a normalization curve specific to that group. After each array was normalized within array, the arrays were then normalized across arrays using the A quantile normalization procedure.

Following normalization each probe within each array was subjected to a smoothing procedure, whereby the probe’s normalized M values were replaced with the median value of all probe normalized M values across all arrays within a 600 bp window. If the number of probes present in the window was less than 3, no value was assigned to that probe. Each probe’s A values were likewise smoothed using the same procedure. Following normalization and smoothing each probe’s M value represents the median intensity difference between vinclozolin lineage and control lineage of a 600 bp window. Significance was assigned to probe differences between lineage and generation samples by calculating the median value of the intensity differences as compared to a normal distribution scaled to the experimental mean and standard deviation of the normalized M. A *Z*-score and *P*-value were computed for each probe from that distribution. The statistical analysis was performed in pairs of comparative IP hybridizations between treatment lineage (T) and control lineage (C) (e.g. T1-C1 and T2-C2; T1-C1 and T3-C3; T2-C2 and T3-C3). In order to assure the reproducibility of the candidates obtained, only the candidates showing significant changes in every one of the paired comparisons were chosen as having a significant change in DNA methylation between each of the experimental group and controls. This is a very stringent approach to select for changes, since it only considers repeated changes in all paired analysis.

The statistically significant differential DNA methylated regions were identified and *P*-value associated with each region presented. Each region of interest was then annotated for gene and CpG content. This list was further reduced to those regions with an average intensity value exceeding 9.5 (log scale) and a CpG density ≥ 1 CpG/100 bp.

### Description of the datasets

The previous methods described above were used to obtain the datasets used in the current study. The different expression datasets collected from the different experiments were female tissue, male tissue [4], Sertoli cell [5], granulosa cell [6] and primordial germ cells [22]. For the epimutation data 776 DMR regions were collected from several experiments investigating transgenerational epigenetic inheritance of disease susceptibility after exposure to the environmental toxicants dioxin, plastics, pesticides, vinclozolin, bisphenol A and jet fuel [3].

### Cluster analysis

For the clustering approach the window size used was 2 Mb (million bases) and this 2 Mb window was shifted 50 kb at a time in overlapped fashion. For each sliding window the number of DMR sites was found. The sliding window is used from the start of the chromosome until the end for each chromosome separately. Once the number of sites had been calculated for each window a *z*-test was used to calculate the *z*-score for each window from which the *p*-value was calculated. Any window with less than 0.05 *p*-value was taken as statistically significant in over-representation of DMR sites. Then finally windows which fall within 50,000 bases (consecutive windows) of each other are merged to create the final cluster. Clusters were calculated for each of the tissue types separately. The R code for this analysis is presented in Additional file 1: Figure S1.

### R code description and supplementary

The R code for the sliding window and the *z*-test is in the Additional file 1: Figure S1 and also is available in the author’s website www.skinner.wsu.edu.

## Abbreviations

DMRs, Differential DNA methylation regions; ECRs, Epigenetic control regions; PGC, Primordial germ cells; FT, Female tissues; MT, Male tissues; BKG, Background; SC, Sertoli cells; GC, Granulosa cells.

## Additional files

Additional file 1: Figure S1. Cluster R-Code. (PDF 52 kb) Additional file 2: Table S1. Male gene clusters. Gene cluster sites with start-end location, and the gene names and classification, and gene start-stop, and types of the tissues for the Male Tissue Array dataset. (PDF 140 kb) Additional file 3: Table S2. Female gene clusters. Gene cluster sites with start-end location of each cluster site, genes in each cluster and classification, and gene start and stop, and types of the tissue for the Female Tissue Array dataset. (PDF 59 kb) Additional file 4: Table S3. Sertoli DMR clusters. DMR clusters with start-end, statistical significance for each DMR with start and stop information for the Sertoli Cell dataset. (PDF 35 kb) Additional file 5: Table S4. Granulosa DMR clusters. DMR clusters with start-end, statistical significance for each DMR with start and stop information for the Granulosa Cell dataset. (PDF 35 kb) Additional file 6: Table S5. Primordial germ cell DMR clusters. DMR clusters with start-end, statistical significance for each DMR with start and stop information regarding the Primordial Germ cell dataset. (PDF 36 kb) Additional file 7: Table S6. Sperm and DMR clusters. DMR clusters with start-end, statistical significance for each DMR with start and stop information regarding clusters for the sperm DMR dataset. (PDF 41 kb)
