# Supplementary material for: Genomic Clustering of differential DNA methylated regions (epimutations) associated with the epigenetic transgenerational inheritance of disease and phenotypic variation
Source: BMC Genomics. 2016 Jun 1;17:418. doi: 10.1186/s12864-016-2748-5 (PMC4888261; doi:10.1186/s12864-016-2748-5)
Supplement: Additional file 1: Figure S1. — Cluster R-Code. (PDF 52 kb) [file 12864_2016_2748_MOESM1_ESM.pdf]

Supplemental Figure S1

```
#########################################################################################################
###########
# Code for clustering
# Input parameters (1) table containing field Choromosome, cSTART, cSTOP, minP and nProbes
#                        (2) windowLength have the sliding window size in million bases
#                        (3) incrementLength have the number of bases the sliding window should be moved
#                                        i.e.
#                                        cluster_table <- Clustering(ctable, 2000000, 50000)
# Output
#                                        table containing Clusters containing fields Chromosome, cSTART, cSTOP and cluster p-value

#########################################################################################################
###########

SlidingWindowCluster <- function(ctable, windowLength, incrementLength)
{
ctable <- ctable[c("Chromosome","cSTART","cSTOP","minP","nProbes")]
ctable <- split(ctable, ctable["Chromosome"])

cmatrix <- matrix(0, nrow=100000, ncol=6, dimnames=list(c(1:100000),c("Chromosome", "cSTART","cSTOP","Count", "Z-score","P-
value")))
index <-0

# loop through each of the chromosomes
for(k in 1: length(names(ctable)))
{
        if(nrow(ctable[[k]])==0)
        {
#       print(paste("no rows on chromosome ", names(ctable[[k]])))
        }else{
                char <- names(ctable)[k]
        #       cat("chromosome :", char )

        #reset counterpatient
                count <- 0
                limit <- as.integer (max(ctable[[k]][,"cSTOP"])/incrementLength)

                                for(i in 1:   limit )
                                {
                                        m =  (i * incrementLength) + windowLength
                                        i = i * incrementLength
                                        count <- 0
                                        # look at the total sites for chromosome k
                                        for(j in 1: nrow(ctable[[k]]))
                                        {
                                                # if the site falls within the million bases
                                                if( (ctable[[k]][j,"cSTART"] > i) & (ctable[[k]][j,"cSTOP"] < m))
                                                {
                                                # then increase counter
                                                count = count +1
                                                }
                                        }
                                        # Enter the repeats in that particular sequence
                                        if(count >= 1)
                                        {
                                        # copy the repeat to "seq" if count is more than 2
                                        seq <- paste("chromosome :", char , "start: ",as.integer(i),
                                        " end: ", as.integer(m), "count: ", as.integer(count) )
                                        cmatrix[index,"Chromosome"] <- as.character(char)
                                        cmatrix[index,"cSTART"] <- as.integer(i)
                                        cmatrix[index,"cSTOP"] <- as.integer(m)
                                        cmatrix[index,"Count"] <- as.integer(count)
                                        index=index+1
                                        }
                                        count <- 0
                                        }
                count = 0
                }
```

```
}

sliding_window <- cmatrix
sliding_window <- sliding_window[1:(head(which(cmatrix[,"Chromosome"]==0),1)-1),]
sliding_window[,"cSTART"] <- as.numeric(as.character(sliding_window[,"cSTART"]))
sliding_window[,"cSTOP"] <- as.numeric(as.character(sliding_window[,"cSTOP"]))
sliding_window[,"Count"] <- as.numeric(as.character(sliding_window[,"Count"]))

# Z-value formula
X <- as.numeric(sliding_window[,"Count"])
meanM <- apply(as.matrix(X, ncol=1), 2, mean)
sdM <- apply(as.matrix(X, ncol=1), 2, sd)

# From the Z-score calculate P-value
Z <- apply( as.matrix(X,ncol=1), 2, function(x)  (x - meanM) / sdM)
P_value <- 2 * pnorm(-abs(Z))

# Insert Z-score and P-value into sliding_window table
sliding_window[,"Z-score"] <- Z
sliding_window[,"P-value"] <- P_value
sliding_window <- sliding_window[order(sliding_window[,"Chromosome"],sliding_window[,"cSTART"]),]
sliding_window <- sliding_window[which(as.numeric(as.character(sliding_window[,"P-value"])) < 0.05),]

clusters <- data.frame(Chromosome=as.character(sliding_window[,"Chromosome"]),
                cSTART=as.integer(as.character(sliding_window[,"cSTART"])),
                cSTOP=as.integer(as.character(sliding_window[,"cSTOP"])),
                Count=as.integer(as.character(sliding_window[,"Count"])),
                Z.score=as.integer(as.character(sliding_window[,"Z-score"])),
                P.value=as.numeric(sliding_window[,"P-value"]))

clusters <- clusters[order(clusters[,"Chromosome"],clusters[,"cSTART"]),]
clusters <- split(clusters,clusters[,"Chromosome"])
#names(clusters)


Seq <- cmatrix <- c()
k <- 0
cluster_p_value <- 0.05
cluster_p_value_first <- 0.05
cluster_p_value_next <- 0.05

for(i in 1: length(names(clusters)))
{
k <- 1
        name <- names(clusters[i])

        limit <- nrow(clusters[[i]])-1

        if(limit==0)
        {
        cluster_start <- as.numeric(clusters[[i]][,"cSTART"])
        cluster_end <- as.numeric(clusters[[i]][,"cSTOP"])
        cluster_p_value <- as.numeric(clusters[[i]][,"P.value"])
        Seq <- data.frame(Chromosome = as.character(name),
        cSTART = as.numeric(cluster_start),
        cSTOP=as.numeric(cluster_end),
        minP = as.numeric(cluster_p_value))
        cmatrix <- rbind(cmatrix,Seq)
        cluster_p_value <- 0
        }

        else
        {

        # go through each row of the chromosome
        for(j in 1: limit)
        {
                # intialize with the first row by default
                if(k==1)
```

```
                {
                m <- clusters[[i]][j,]
                cluster_start <- as.numeric(clusters[[i]][j,"cSTART"])
                cluster_p_value <- as.numeric(clusters[[i]][j,"P.value"])
                k <- 0
                }

        start1 <- clusters[[i]][j,"cSTOP"]
        more <- j+1
        start2 <- clusters[[i]][more,2]
                if( (start2 - start1) > 1000000)
                {
                cluster_end <- as.numeric(clusters[[i]][j,"cSTOP"])

                cluster_p_value_next <- as.numeric(clusters[[i]][j,"P.value"])

                        if(as.numeric(cluster_p_value_next) < as.numeric(cluster_p_value_first))
                        {
                        cluster_p_value <- as.numeric(cluster_p_value_next)
                        }
                        else
                        {
                        cluster_p_value <- as.numeric(cluster_p_value_first)
                        }

                Seq <- data.frame(Chromosome = as.character(name),
                cSTART = as.numeric(cluster_start),
                cSTOP=as.numeric(cluster_end),
                minP = as.numeric(cluster_p_value))
                cmatrix <- rbind(cmatrix,Seq)
                cluster_start <- as.numeric(clusters[[i]][more,"cSTART"])

                }
                        # compare the temp (cluster_p_value) with each (cluster_p_value_next)
                        cluster_p_value_next <- as.numeric(clusters[[i]][j,"P.value"])

                        # save the lowest p-value for the cluster
                        if(as.numeric(cluster_p_value_next) < as.numeric(cluster_p_value))
                        {
                        cluster_p_value <- as.numeric(cluster_p_value_next)
                        }
                        else
                        {
                        cluster_p_value <- as.numeric(cluster_p_value)
                        }

                end1 <- clusters[[i]][j,"cSTOP"]
                end2 <- clusters[[i]][j+1,"cSTART"]
                if( (end2 -end1) < 1000000)
                {
                        if(j==limit)
                        {
                        cluster_end <-        as.numeric(clusters[[i]][j,"cSTOP"])
                        Seq <- data.frame(Chromosome = as.character(name),
                        cSTART = as.numeric(cluster_start),
                        cSTOP=as.numeric(cluster_end),
                        minP = as.numeric(cluster_p_value))
                        cmatrix <- rbind(cmatrix,Seq)
                        }
                }
        }
        cluster_p_value <- 0
        }
}


return (cmatrix)
}
```
