# Supplementary material for: Genomic Clustering of differential DNA methylated regions (epimutations) associated with the epigenetic transgenerational inheritance of disease and phenotypic variation
Source: BMC Genomics. 2016 Jun 1;17:418. doi: 10.1186/s12864-016-2748-5 (PMC4888261; doi:10.1186/s12864-016-2748-5)
Supplement: Additional file 2: Table S1. — Male gene clusters. Gene cluster sites with start-end location, and the gene names and classification, and gene start-stop, and types of the tissues for the Male Tissue Array dataset. (PDF 140 kb) [file 12864_2016_2748_MOESM2_ESM.pdf]

Supplemental Table S1 **Male Tissue Gene Cluster**

Cluster Chromosome	ClusterStart	ClusterEnd	Gene Name	Gene Classification	Gene cSTART	Gene cSTOP	Tissue Type
chr1	78400000	86500000	Ltv1	Development	7956276	7968981	M.SV
chr1	78400000	86500000	Qpctl	Metabolism	78500461	78508517	M.Liv
chr1	78400000	86500000	Snrpd2	Translation	78509142	78511991	M.Tst
chr1	78400000	86500000	Ercc1	Epigenetics	78709565	78722422	M.Prst
chr1	78400000	86500000	LOC686781	NA	78733982	78896293	M.Prst
chr1	78400000	86500000	Bloc1s3	Metabolism	78860378	78860966	M.Kdn
chr1	78400000	86500000	Trappc6a	Metabolism	78862235	78869042	M.Kdn
chr1	78400000	86500000	Clptm1	Metabolism	78938921	78970671	M.Prst
chr1	78400000	86500000	Pvrl2	Receptors	79021827	79059686	M.Prst
chr1	78400000	86500000	Ceacam6	NA	80110384	80129016	M.Kdn
chr1	78400000	86500000	Znf574	NA	80390353	80395599	M.Tst
chr1	78400000	86500000	Gsk3a	Signaling	80505517	80514138	M.Prst
chr1	78400000	86500000	---	NA	80505564	80505988	M.Prst
chr1	78400000	86500000	Pafah1b3	Metabolism	80570160	80572686	M.Kdn
chr1	78400000	86500000	Lipe	Metabolism	80663791	80682480	M.SV
chr1	78400000	86500000	Atp5sl	Metabolism	80827838	80833767	M.SV
chr1	78400000	86500000	Bckdha	Metabolism	80837906	80866672	M.Tst
chr1	78400000	86500000	Axl	Receptor	80964751	80994323	M.Liv
chr1	78400000	86500000	Sdccag1	NA	82120277	82122428	M.Kdn
chr1	78400000	86500000	Blvrb	Metabolism	82529086	82546598	M.SV
chr1	78400000	86500000	Pld3	Metabolism	82612829	82627294	M.Prst
chr1	78400000	86500000	Psmc4	Proteolysis	83151146	83159470	M.Kdn
chr1	78400000	86500000	Eid2	NA	83349864	83350580	M.Kdn
chr1	78400000	86500000	LOC687295	Metabolism	83398721	83400789	M.Tst
chr1	78400000	86500000	Zfp36	Transcription	83486647	83489123	M.SV
chr1	78400000	86500000	Zfp36	Transcription	83486647	83489123	M.Prst
chr1	78400000	86500000	Zfp36	Transcription	83486647	83489123	M.Kdn
chr1	78400000	86500000	Gmfg	Signaling	83546798	83557015	M.Prst
chr1	78400000	86500000	RGD1303117	NA	84284332	84289134	M.Prst
chr1	78400000	86500000	Yif1b	Development	84373710	84383874	M.Prst
chr1	78400000	86500000	Sipa1l3	Signaling	84456418	84509587	M.Prst
chr1	78400000	86500000	Sipa1l3	Signaling	84522451	84522608	M.Prst
chr1	78400000	86500000	Sipa1l3	Signaling	84526515	84526689	M.Prst
chr1	78400000	86500000	Sipa1l3	Signaling	84536782	84536912	M.Prst
chr1	78400000	86500000	Sipa1l3	Signaling	84538108	84540559	M.Prst
chr1	78400000	86500000	RGD1562079	Miscellaneous	85268360	85268716	M.Kdn
chr1	78400000	86500000	Tyrobp	Receptor	85365371	85369227	M.Prst
chr1	78400000	86500000	Tyrobp	Receptor	85365371	85369227	M.Kdn
chr1	78400000	86500000	Aplp1	Transcription	85390513	85399802	M.Prst
chr1	78400000	86500000	Polr2i	Transcription	85489091	85490390	M.Kdn
chr1	78400000	86500000	Tmem147	Receptor	85771683	85773503	M.Kdn
chr1	78400000	86500000	Gramd1a	Unknown	86175390	86196895	M.SV
chr1	93350000	97450000	RGD1564800	NA	93542062	93542430	M.Prst
chr1	93350000	97450000	Siglec5	Receptor	93734935	93742543	M.Liv
chr1	93350000	97450000	Atpbd3	NA	94085739	94088431	M.Prst
chr1	93350000	97450000	Klk1l	NA	94624828	94628822	M.Kdn
chr1	93350000	97450000	MGC93975	NA	94967722	94974041	M.Prst
chr1	93350000	97450000	MGC93975	NA	94967722	94974041	M.Kdn
chr1	93350000	97450000	Napsa	Protease	95050769	95062774	M.Liv
chr1	93350000	97450000	Myh14	Cytoskeleton	95081948	95146455	M.Prst
chr1	93350000	97450000	Pnkp	Signaling	95330455	95335468	M.Kdn
chr1	93350000	97450000	Ptov1	Transcription	95335896	95342760	M.Prst

chr1	93350000	97450000	Med25	Transcription	95350225	95364484	M.Prst
chr1	93350000	97450000	Irf3	Transcription	95470284	95525050	M.Kdn
chr1	93350000	97450000	Rras	Signaling	95490697	95495452	M.Kdn
chr1	93350000	97450000	Aldh16a1	Metabolism	95553561	95630798	M.Kdn
chr1	93350000	97450000	ENSRNOT00000053402	NA	95601754	95601835	M.Tst
chr1	93350000	97450000	ENSRNOT00000052978	NA	95602277	95602357	M.Tst
chr1	93350000	97450000	Cd37	Signaling	95709954	95715614	M.Prst
chr1	93350000	97450000	Trpm4	Transport	95773700	95803496	M.Prst
chr1	93350000	97450000	Snrp70	NA	95847853	95868392	M.Kdn
chr1	93350000	97450000	Sphk2	Signaling	96178615	96184993	M.Prst
chr1	93350000	97450000	Nomo1	Protease	96528665	96579464	M.Prst
chr1	93350000	97450000	LOC494539	NA	97324367	97324774	M.Kdn
chr1	109400000	113350000	ENSRNOT00000053882	NA	111288045	111288137	M.Tst
chr1	109400000	113350000	ENSRNOT00000053882	NA	111288045	111288137	M.Prst
chr1	109400000	113350000	ENSRNOT00000054014	NA	111338314	111338406	M.Kdn
chr1	109400000	113350000	ENSRNOT00000053184	NA	111346992	111347084	M.Tst
chr1	109400000	113350000	ENSRNOT00000052564	NA	111349177	111349269	M.Tst
chr1	109400000	113350000	ENSRNOT00000052564	NA	111349177	111349269	M.Prst
chr1	109400000	113350000	ENSRNOT00000053117	NA	111353374	111353466	M.Tst
chr1	109400000	113350000	ENSRNOT00000052661	NA	111358225	111358317	M.Tst
chr1	109400000	113350000	ENSRNOT00000053882	NA	111360802	111360894	M.Tst
chr1	109400000	113350000	ENSRNOT00000053882	NA	111360802	111360894	M.Prst
chr1	109400000	113350000	ENSRNOT00000052661	NA	111363398	111363490	M.Tst
chr1	109400000	113350000	ENSRNOT00000053936	NA	111369407	111369499	M.Tst
chr1	109400000	113350000	ENSRNOT00000053882	NA	111371982	111372074	M.Tst
chr1	109400000	113350000	ENSRNOT00000053882	NA	111371982	111372074	M.Prst
chr1	109400000	113350000	ENSRNOT00000053882	NA	111374556	111374648	M.Tst
chr1	109400000	113350000	ENSRNOT00000053882	NA	111374556	111374648	M.Prst
chr1	109400000	113350000	ENSRNOT00000053019	NA	111379836	111379928	M.Tst
chr1	109400000	113350000	ENSRNOT00000053882	NA	111386349	111386441	M.Tst
chr1	109400000	113350000	ENSRNOT00000053882	NA	111386349	111386441	M.Prst
chr1	109400000	113350000	ENSRNOT00000053882	NA	111388918	111389010	M.Tst
chr1	109400000	113350000	ENSRNOT00000053882	NA	111388918	111389010	M.Prst
chr1	109400000	113350000	ENSRNOT00000053882	NA	111391497	111391589	M.Tst
chr1	109400000	113350000	ENSRNOT00000053882	NA	111391497	111391589	M.Prst
chr1	109400000	113350000	ENSRNOT00000053974	NA	111396632	111396724	M.Tst
chr1	109400000	113350000	ENSRNOT00000053882	NA	111399841	111399933	M.Tst
chr1	109400000	113350000	ENSRNOT00000053882	NA	111399841	111399933	M.Prst
chr1	109400000	113350000	ENSRNOT00000053934	NA	111450980	111451072	M.Tst
chr1	109400000	113350000	ENSRNOT00000053882	NA	111456841	111456933	M.Tst
chr1	109400000	113350000	ENSRNOT00000053882	NA	111456841	111456933	M.Prst
chr1	109400000	113350000	ENSRNOT00000052507	NA	111459481	111459573	M.Tst
chr1	109400000	113350000	ENSRNOT00000052993	NA	111464457	111464549	M.Kdn
chr1	109400000	113350000	ENSRNOT00000053882	NA	111467045	111467137	M.Tst
chr1	109400000	113350000	ENSRNOT00000053882	NA	111467045	111467137	M.Prst
chr1	109400000	113350000	ENSRNOT00000053081	NA	111469638	111469730	M.Prst
chr1	109400000	113350000	ENSRNOT00000053855	NA	111472244	111472335	M.Tst
chr1	184850000	187650000	Xpo6	Transport	185180272	185269588	M.Tst
chr1	184850000	187650000	Rabep2	Signaling	185536967	185553191	M.Prst
chr1	184850000	187650000	Eif3c	Translation	185691630	185709597	M.SV
chr1	184850000	187650000	Coro1a	Cytoskeleton	185852741	185857715	M.Kdn
chr1	184850000	187650000	Ccdc95	NA	186030833	186041424	M.SV
chr1	184850000	187650000	Cdipt	Metabolism	186153278	186157417	M.Tst
chr1	184850000	187650000	Tbc1d10b	Signaling	186457439	186468587	M.Prst
chr1	184850000	187650000	Xtp3tpa	NA	186519952	186523348	M.Tst
chr1	184850000	187650000	Prr14	Metabolism	186728878	186776101	M.Tst

chr1	184850000	187650000	ENSRNOT00000053325	NA	186812653	186812786	M.Tst
chr1	184850000	187650000	Rnf40	Proteolysis	186876103	186890731	M.Prst
chr1	184850000	187650000	Bcl7c	Transcription	186993655	186997355	M.Kdn
chr1	200800000	203450000	Rnh1	Translation	201358638	201371015	M.Kdn
chr1	200800000	203450000	Irf7	Transcription	201456689	201459756	M.Prst
chr1	200800000	203450000	Tmem80	Miscellaneous	201524983	201533594	M.Hrt
chr1	200800000	203450000	Tspan4	Cytoskeleton	201661383	201681078	M.Kdn
chr1	200800000	203450000	Tspan4	Cytoskeleton	201661383	201681078	M.Hrt
chr1	200800000	203450000	Tollip	Immune	201997982	202029669	M.Prst
chr1	200800000	203450000	Lsp1	Cytoskeleton	202707305	202741026	M.Prst
chr1	200800000	203450000	Lsp1	Cytoskeleton	202707305	202741026	M.Liv
chr1	200800000	203450000	Lsp1	Cytoskeleton	202707305	202741026	M.Kdn
chr1	200800000	203450000	Mrpl23	Translation	202780065	202787834	M.Kdn
chr1	200800000	203450000	Tssc4	Transcription	203344569	203346200	M.Prst
chr1	204750000	215150000	Med23	Transcription	20999942	21060567	M.Tst
chr1	204750000	215150000	Enpp1	Signaling	21223674	21287411	M.Tst
chr1	204750000	215150000	Ctgf	Growth Factor	21327099	21330215	M.Kdn
chr1	204750000	215150000	Ppfia1	Signaling	204908188	204984408	M.Tst
chr1	204750000	215150000	Ppfia1	Signaling	204951084	204951332	M.Prst
chr1	204750000	215150000	Ppfia1	Signaling	204951084	204951332	M.Kdn
chr1	204750000	215150000	Ccnd1	Cell cycle	205357283	205366810	M.Prst
chr1	204750000	215150000	Cpt1a	Metabolism	205852746	205913024	M.Prst
chr1	204750000	215150000	Chka	Signaling	206368963	206418527	M.Liv
chr1	204750000	215150000	Ndufs8	Metabolism	206433668	206436543	M.Tst
chr1	204750000	215150000	Unc93b1	Immune	206460327	206471241	M.Kdn
chr1	204750000	215150000	Aip	Protein binding	206697248	206704462	M.Tst
chr1	204750000	215150000	Tmem134	Miscellaneous	206708922	206714409	M.Kdn
chr1	204750000	215150000	Rpl35a	Translation	206730060	206730392	M.Tst
chr1	204750000	215150000	LOC100361529	NA	206778858	206785229	M.Tst
chr1	204750000	215150000	Rce1	Protease	207213688	207216827	M.Prst
chr1	204750000	215150000	Rbm14	Translation	207411506	207421695	M.Tst
chr1	204750000	215150000	Zdhhc24	Transcription	207493087	207499194	M.Prst
chr1	204750000	215150000	Slc29a2	Transport	207643719	207650360	M.Tst
chr1	204750000	215150000	B3gnt1	Metabolism	207659001	207661260	M.Kdn
chr1	204750000	215150000	Yif1	NA	207711336	207715338	M.Kdn
chr1	204750000	215150000	Eif1ad	Translation	208010510	208015810	M.Kdn
chr1	204750000	215150000	RGD1562657	NA	208214415	208217148	M.Prst
chr1	204750000	215150000	Rela	Transcription	208262669	208273148	M.SV
chr1	204750000	215150000	Pcnxl3	Development	208288930	208311240	M.Tst
chr1	204750000	215150000	Mtvr2	NA	208355217	208356772	M.Kdn
chr1	204750000	215150000	Ltbp3	Signaling	208366405	208385177	M.Hrt
chr1	204750000	215150000	BC090353	NA	208428092	208428255	M.Prst
chr1	204750000	215150000	BC090353	NA	208428092	208428255	M.Kdn
chr1	204750000	215150000	Frmd8	Signaling	208489337	208509947	M.Kdn
chr1	204750000	215150000	Dpf2	Transcription	208530922	208544954	M.Tst
chr1	204750000	215150000	Syvn1	Protease	208684467	208690975	M.Prst
chr1	204750000	215150000	Cdc42bpg	Signaling	209084212	209111593	M.Prst
chr1	204750000	215150000	Sf1	Translation	209146188	209159459	M.Tst
chr1	204750000	215150000	RGD1566262	NA	209405376	209405903	M.Prst
chr1	204750000	215150000	RGD1566262	NA	209405376	209405903	M.Kdn
chr1	204750000	215150000	Esrra	Receptors	209587682	209597677	M.Liv
chr1	204750000	215150000	Trpt1	Translation	209669953	209672820	M.Prst
chr1	204750000	215150000	Otub1	Protease	209871581	209886083	M.Tst
chr1	204750000	215150000	Otub1	Protease	209871581	209886083	M.Kdn
chr1	204750000	215150000	Cox8a	Electron transport	209886132	209888462	M.Kdn
chr1	204750000	215150000	Mark2	Signaling	209958902	210022099	M.Prst

chr1	204750000	215150000	RGD1560108	NA	210107906	210108290	M.SV
chr1	204750000	215150000	Rtn3	Transport	210109534	210166035	M.Prst
chr1	204750000	215150000	Slc3a2	Metabolism	211375889	211390313	M.Prst
chr1	204750000	215150000	ENSRNOT00000054147	NA	211392417	211392480	M.Tst
chr1	204750000	215150000	ENSRNOT00000052494	NA	211392650	211392718	M.Kdn
chr1	204750000	215150000	ENSRNOT00000052494	NA	211392650	211392718	M.Hrt
chr1	204750000	215150000	ENSRNOT00000053552	NA	211393150	211393275	M.Tst
chr1	204750000	215150000	Nxf1	Transcription	211428457	211441798	M.Tst
chr1	204750000	215150000	Nxf1	Transcription	211428457	211441798	M.Kdn
chr1	204750000	215150000	Tmem223	NA	211441885	211443098	M.Tst
chr1	204750000	215150000	Tmem223	NA	211441885	211443098	M.Kdn
chr1	204750000	215150000	Ints5	Miscellaneous	211568313	211573094	M.Prst
chr1	204750000	215150000	Ganab	Metabolism	211573302	211594011	M.Prst
chr1	204750000	215150000	Eef1g	Translation	211640225	211651560	M.Kdn
chr1	204750000	215150000	LOC100362601	NA	212228789	212232842	M.Tst
chr1	204750000	215150000	Rab3il1	Signaling	212463114	212482280	M.Liv
chr1	204750000	215150000	Cpsf7	Translation	213009572	213032629	M.Tst
chr1	204750000	215150000	Cpsf7	Translation	213009572	213032629	M.Kdn
chr1	204750000	215150000	ENSRNOT00000053169	NA	213047106	213047223	M.Kdn
chr1	204750000	215150000	ENSRNOT00000053169	NA	213047106	213047223	M.Hrt
chr1	204750000	215150000	Ddb1	DNA repair	213095356	213120520	M.Prst
chr1	204750000	215150000	Vps37c	Proteolysis	213202291	213208896	M.Prst
chr1	204750000	215150000	Slc15a3	Transport	213341322	213356103	M.SV
chr1	204750000	215150000	Tmem109	Miscellaneous	213368573	213379148	M.Liv
chr1	204750000	215150000	Ms4a11	NA	214045392	214057578	M.SV
chr1	204750000	215150000	Ms4a11	NA	214045392	214057578	M.Prst
chr1	204750000	215150000	Ms4a11	NA	214045392	214057578	M.Liv
chr1	204750000	215150000	Ms4a6a	Receptor	214162552	214176200	M.Prst
chr1	204750000	215150000	Ms4a6b	Receptors	214208053	214219878	M.SV
chr1	204750000	215150000	ENSRNOT00000069284	NA	214655070	214655193	M.Tst
chr1	204750000	215150000	LOC100363176	NA	214863977	214869264	M.Tst
chr2	180150000	183600000	RGD1303130	Development	180438070	180441660	M.Prst
chr2	180150000	183600000	Slc25a44	Binding Protein	180514428	180526461	M.Prst
chr2	180150000	183600000	Sema4a	Signaling	180552405	180573625	M.Prst
chr2	180150000	183600000	Lmna	Cytoskeleton	180595722	180616354	M.Kdn
chr2	180150000	183600000	Dap3	Translation	180987261	181014360	M.SV
chr2	180150000	183600000	Krtcap2	Cytoskeleton	181417465	181421069	M.SV
chr2	180150000	183600000	Lenep	Cytoskeleton	181598076	181607196	M.Prst
chr2	180150000	183600000	RGD1561797	NA	181612977	181616186	M.Tst
chr2	180150000	183600000	Pygo2	Epigenetics	181633028	181633349	M.Prst
chr2	180150000	183600000	Il6ra	Receptor	182078051	182128147	M.Liv
chr2	180150000	183600000	LOC685152	Transport	182166741	182166885	M.Hrt
chr2	180150000	183600000	RGD1564171	Miscellaneous	182287947	182290740	M.SV
chr2	180150000	183600000	Rps27	Translation	182451530	182452619	M.Tst
chr2	180150000	183600000	Dennd4b	Signaling	182507256	182522690	M.Kdn
chr2	180150000	183600000	Ints3	Miscellaneous	182650173	182702440	M.Tst
chr2	180150000	183600000	Ints3	Miscellaneous	182650173	182702440	M.Prst
chr2	180150000	183600000	S100a6	Signaling	182895080	182896305	M.Tst
chr2	180150000	183600000	S100a6	Signaling	182895080	182896305	M.Kdn
chr2	188650000	192550000	mrpl9	Translation	189417391	189422114	M.Tst
chr2	188650000	192550000	LOC100363361	Transcription	189907124	189915547	M.Tst
chr2	188650000	192550000	LOC100363361	Transcription	189907124	189915547	M.Prst
chr2	188650000	192550000	Vps72	Transcription	190012907	190024404	M.Prst
chr2	188650000	192550000	Lass2	NA	190224045	190233351	M.Tst
chr2	188650000	192550000	Setdb1	Epigenetics	190232228	190264289	M.Kdn
chr2	188650000	192550000	Ctsk	Protease	190394854	190405668	M.Hrt

chr2	188650000	192550000	Ctss	Protease	190422634	190466877	M.Liv
chr2	188650000	192550000	Tars2	Metabolism	190530782	190547829	M.Prst
chr2	188650000	192550000	Prpf3	Translation	190617892	190642728	M.Liv
chr2	188650000	192550000	LOC690012	NA	190666850	190676146	M.Liv
chr2	188650000	192550000	Hist2h3c2	Epigenetics	191043639	191044191	M.Kdn
chr2	188650000	192550000	Hist2h3c2	Epigenetics	191053229	191053801	M.Kdn
chr2	188650000	192550000	Hist2h2bb	Epigenetics	191082482	191089577	M.SV
chr2	188650000	192550000	Fcgr1a	Immune	191095027	191103953	M.Prst
chr2	188650000	192550000	Fcgr1a	Immune	191095027	191103953	M.Liv
chr2	188650000	192550000	Txnip	Transcription	191356954	191360761	M.SV
chr2	188650000	192550000	Txnip	Transcription	191356954	191360761	M.Liv
chr2	188650000	192550000	Acp6	Signaling	191942045	191964330	M.Prst
chr3	144750000	147750000	RGD1559743	NA	14651432	14651972	M.Kdn
chr3	144750000	147750000	ENSRNOT00000069216	NA	145372837	145372929	M.Hrt
chr3	144750000	147750000	Map1lc3a	Cytoskeleton	145754265	145760532	M.Prst
chr3	144750000	147750000	Ncoa6	Transcription	145886880	145930308	M.Tst
chr3	144750000	147750000	---	NA	145941387	145941635	M.Kdn
chr3	144750000	147750000	Trpc4ap	Development	146139484	146206622	M.Prst
chr3	144750000	147750000	Edem2	Metabolism	146215241	146241062	M.Hrt
chr3	144750000	147750000	Ergic3	Golgi apparatus	146416480	146426322	M.Tst
chr3	144750000	147750000	Ergic3	Golgi apparatus	146416480	146426322	M.Prst
chr3	144750000	147750000	Rbm39	Translation	146554521	146587661	M.Kdn
chr3	144750000	147750000	Scand1	Transcription	146710193	146710650	M.Kdn
chr3	144750000	147750000	Ndrg3	Transcription	147403379	147422617	M.Prst
chr4	75400000	77850000	FQ223410	NA	75915531	75915630	M.Tst
chr4	75400000	77850000	ENSRNOT00000053328	NA	75918789	75918898	M.Tst
chr4	75400000	77850000	Pdia4	Metabolism	75929252	75948299	M.SV
chr4	75400000	77850000	Zfp212	Transcription	76049107	76060970	M.Prst
chr4	75400000	77850000	Znf746	NA	76184770	76209237	M.Prst
chr4	75400000	77850000	Repin1	Transcription	76688429	76690141	M.SV
chr4	75400000	77850000	Tmem176a	Miscellaneous	76920036	76923598	M.Kdn
chr4	75400000	77850000	Gpnmb	Cytoskeleton	77161352	77182963	M.Liv
chr4	75400000	77850000	---	NA	77390402	77390592	M.Prst
chr4	75400000	77850000	---	NA	77390402	77390592	M.Kdn
chr4	75400000	77850000	---	NA	77390402	77390592	M.Hrt
chr4	75400000	77850000	Ccdc126	Metabolism	77445717	77466089	M.Hrt
chr5	136750000	138950000	Ccdc17	Transcription	136879831	136883445	M.Kdn
chr5	136750000	138950000	Mmachc	Development	136995878	137001362	M.Tst
chr5	136750000	138950000	Mmachc	Development	136995878	137001362	M.Prst
chr5	136750000	138950000	Zswim5	Transcription	137177685	137295481	M.Kdn
chr5	136750000	138950000	ENSRNOT00000056936	NA	137190345	137190655	M.Tst
chr5	136750000	138950000	GENSCAN00000021509	NA	137468957	137469035	M.Kdn
chr5	136750000	138950000	ENSRNOT00000052542	NA	137509717	137509832	M.Tst
chr5	136750000	138950000	BC105775	NA	137577433	137577758	M.SV
chr5	136750000	138950000	Kdm4a	Epigenetics	138603000	138667807	M.Kdn
chr5	136750000	138950000	Ptprf	Signaling	138670952	138739002	M.Prst
chr5	136750000	138950000	RGD1308616	NA	138854045	138854211	M.Prst
chr5	163750000	167450000	ENSRNOT00000054574	NA	164356525	164356629	M.Kdn
chr5	163750000	167450000	LOC691261	NA	164456648	164483642	M.Kdn
chr5	163750000	167450000	LOC500584	NA	164504972	164515847	M.Kdn
chr5	163750000	167450000	LOC691280	NA	164913570	164931588	M.Tst
chr5	163750000	167450000	Mthfr	Metabolism	165112850	165126885	M.Prst
chr5	163750000	167450000	Rpl35a	Translation	165189350	165189682	M.Tst
chr5	163750000	167450000	Mad2l2	Transcription	165218362	165222828	M.Prst
chr5	163750000	167450000	Fbxo6	Transcription	165222858	165226423	M.Kdn
chr5	163750000	167450000	Ubiad1	Metabolism	165516860	165526571	M.Prst

chr5	163750000	167450000	ENSRNOT00000053105	NA	165705419	165705554	M.Tst
chr5	163750000	167450000	Dffa	Miscellaneous	166179130	166192029	M.Prst
chr5	163750000	167450000	Kif1b	Cytoskeleton	166249002	166381782	M.Prst
chr5	163750000	167450000	Ube4b	Protease	166407096	166508391	M.Prst
chr5	163750000	167450000	Clstn1	Extracellular matrix	166671079	166734905	M.Prst
chr5	163750000	167450000	Tmem201	NA	166810397	166830271	M.Prst
chr5	163750000	167450000	LOC691431	NA	166836753	166861368	M.Kdn
chr5	163750000	167450000	Spsb1	Proteolysis	167012678	167025639	M.Kdn
chr5	163750000	167450000	Gpr157	Receptor	167197510	167223745	M.Prst
chr6	132300000	136300000	LOC690965	NA	132572639	132641171	M.Prst
chr6	132300000	136300000	Slc25a29	Binding Protein	133166812	133177600	M.Prst
chr6	132300000	136300000	Wars	Metabolism	133200866	133232048	M.Prst
chr6	132300000	136300000	rno-mir-341	NA	134203518	134203613	M.Kdn
chr6	132300000	136300000	ENSRNOT00000053070	NA	134244770	134244850	M.Prst
chr6	132300000	136300000	FQ213989	NA	134252751	134252836	M.Prst
chr6	132300000	136300000	FQ213989	NA	134254537	134254622	M.Prst
chr6	132300000	136300000	ENSRNOT00000054133	NA	134256322	134256416	M.Prst
chr6	132300000	136300000	AB014881	NA	134258110	134258200	M.Prst
chr6	132300000	136300000	ENSRNOT00000053832	NA	134259001	134259092	M.Prst
chr6	132300000	136300000	AB014881	NA	134260788	134260882	M.Prst
chr6	132300000	136300000	AB014881	NA	134263473	134263557	M.Prst
chr6	132300000	136300000	AB014879	NA	134265258	134265338	M.Prst
chr6	132300000	136300000	AB014879	NA	134269705	134269791	M.Prst
chr6	132300000	136300000	AB014879	NA	134270805	134270885	M.Prst
chr6	132300000	136300000	AB014878	NA	134271691	134271778	M.Prst
chr6	132300000	136300000	AB014879	NA	134272581	134272670	M.Prst
chr6	132300000	136300000	AB014879	NA	134273476	134273556	M.Prst
chr6	132300000	136300000	AB014878	NA	134274363	134274453	M.Prst
chr6	132300000	136300000	AB014879	NA	134275256	134275342	M.Prst
chr6	132300000	136300000	AB014879	NA	134276149	134276229	M.Prst
chr6	132300000	136300000	AB014877	NA	134278112	134278199	M.Prst
chr6	132300000	136300000	AB014879	NA	134279897	134279977	M.Prst
chr6	132300000	136300000	AB014881	NA	134280783	134280871	M.Prst
chr6	132300000	136300000	AB014879	NA	134281675	134281761	M.Prst
chr6	132300000	136300000	AB014879	NA	134282568	134282648	M.Prst
chr6	132300000	136300000	AB014879	NA	134286979	134287068	M.Prst
chr6	132300000	136300000	AB014879	NA	134287876	134287956	M.Prst
chr6	132300000	136300000	AB014878	NA	134288761	134288850	M.Prst
chr6	132300000	136300000	AB014877	NA	134290666	134290752	M.Prst
chr6	132300000	136300000	AB014879	NA	134291560	134291640	M.Prst
chr6	132300000	136300000	AB014878	NA	134292447	134292535	M.Prst
chr6	132300000	136300000	AB014877	NA	134293338	134293426	M.Prst
chr6	132300000	136300000	ENSRNOT00000054021	NA	134294545	134294637	M.Prst
chr6	132300000	136300000	AB014879	NA	134295442	134295530	M.Prst
chr6	132300000	136300000	AB014879	NA	134296338	134296418	M.Prst
chr6	132300000	136300000	ENSRNOT00000054054	NA	134297224	134297310	M.Prst
chr6	132300000	136300000	AB014878	NA	134298116	134298203	M.Prst
chr6	132300000	136300000	AB014879	NA	134299010	134299090	M.Prst
chr6	132300000	136300000	AB014879	NA	134300789	134300869	M.Prst
chr6	132300000	136300000	AB014878	NA	134301675	134301762	M.Prst
chr6	132300000	136300000	ENSRNOT00000053573	NA	134302564	134302651	M.Prst
chr6	132300000	136300000	AB014879	NA	134303458	134303538	M.Prst
chr6	132300000	136300000	AB014878	NA	134304344	134304431	M.Prst
chr6	132300000	136300000	AB014878	NA	134305803	134305890	M.Prst
chr6	132300000	136300000	AB014877	NA	134306693	134306778	M.Prst
chr6	132300000	136300000	AB014877	NA	134307586	134307666	M.Prst

chr6	132300000	136300000	AB014878	NA	134308472	134308560	M.Prst
chr6	132300000	136300000	AB014879	NA	134309363	134309449	M.Prst
chr6	132300000	136300000	AB014879	NA	134310258	134310338	M.Prst
chr6	132300000	136300000	AB014877	NA	134311145	134311237	M.Prst
chr6	132300000	136300000	AB014879	NA	134313000	134313080	M.Prst
chr6	132300000	136300000	FQ212998	NA	134313886	134313979	M.Prst
chr6	132300000	136300000	AB014879	NA	134315681	134315761	M.Prst
chr6	132300000	136300000	AB014879	NA	134317544	134317624	M.Prst
chr6	132300000	136300000	AB014879	NA	134319325	134319405	M.Prst
chr6	132300000	136300000	ENSRNOT00000054054	NA	134320214	134320300	M.Prst
chr6	132300000	136300000	AB014877	NA	134321101	134321193	M.Prst
chr6	132300000	136300000	AB014879	NA	134321999	134322079	M.Prst
chr6	132300000	136300000	FQ213989	NA	134322885	134322975	M.Prst
chr6	132300000	136300000	AB014879	NA	134323783	134323863	M.Prst
chr6	132300000	136300000	AB014879	NA	134326456	134326536	M.Prst
chr6	132300000	136300000	Mir667	NA	134400500	134400591	M.Kdn
chr6	132300000	136300000	Tecpr2	Miscellaneous	135619756	135709735	M.Prst
chr6	132300000	136300000	LOC100363014	NA	135783794	135858490	M.Prst
chr6	137400000	139850000	LOC500726	NA	137707120	137730829	M.Prst
chr6	137400000	139850000	LOC691616	NA	137890464	137892084	M.SV
chr6	137400000	139850000	LOC691616	NA	137890464	137892084	M.Prst
chr6	137400000	139850000	Jag2	Receptor	137909705	137931131	M.Prst
chr6	137400000	139850000	Nudt14	Metabolism	137935991	137942943	M.Tst
chr6	137400000	139850000	Mta1	Transcription	138118468	138143983	M.Prst
chr6	137400000	139850000	Adam6	Receptor	138855420	138890431	M.Prst
chr6	137400000	139850000	Ighg	NA	138941043	138942176	M.Prst
chr6	137400000	139850000	ENSRNOT00000048037	NA	139361659	139362149	M.Prst
chr6	137400000	139850000	LOC678701	NA	139397563	139398544	M.Prst
chr6	137400000	139850000	IgG-2a	NA	139764413	139765553	M.Prst
chr6	137400000	139850000	IgG-2a	NA	139773176	139773676	M.Prst
chr6	137400000	139850000	FQ218314	NA	139813361	139814238	M.Kdn
chr7	112300000	116400000	Mbrl	Signaling	11236950	11244044	M.Prst
chr7	112300000	116400000	Wdr18	Miscellaneous	11253373	11261805	M.Tst
chr7	112300000	116400000	RGD1307067	NA	11301479	11309971	M.Kdn
chr7	112300000	116400000	Med16	Transcription	11311092	11323250	M.Prst
chr7	112300000	116400000	RGD1359378	NA	112835301	112838479	M.Kdn
chr7	112300000	116400000	Ly6e	Immune	113151828	113155521	M.Liv
chr7	112300000	116400000	Ly6c	Immune	113178690	113182136	M.Prst
chr7	112300000	116400000	Ly6c	Immune	113178690	113182136	M.Liv
chr7	112300000	116400000	Gsdmd	Apoptosis	113813191	113837009	M.Kdn
chr7	112300000	116400000	Tsta3	Metabolism	113929477	113933642	M.Tst
chr7	112300000	116400000	Scrib	Miscellaneous	114077733	114100794	M.SV
chr7	112300000	116400000	Scrib	Miscellaneous	114077733	114100794	M.Prst
chr7	112300000	116400000	Siahbp1	NA	114101189	114112125	M.Prst
chr7	112300000	116400000	Plec1	NA	114202744	114262385	M.Prst
chr7	112300000	116400000	Exosc4	Metabolism	114374951	114377370	M.Kdn
chr7	112300000	116400000	rCG_59505	NA	114429792	114485051	M.Prst
chr7	112300000	116400000	ENSRNOT00000056375	NA	114490914	114500210	M.Prst
chr7	112300000	116400000	Bop1	Translation	114500155	114524030	M.Prst
chr7	112300000	116400000	Bop1	Translation	114500155	114524030	M.Liv
chr7	112300000	116400000	Bop1	Translation	114500155	114524030	M.Kdn
chr7	112300000	116400000	Mfsd3	Metabolism	114750871	114752873	M.Kdn
chr7	112300000	116400000	RGD1309808	Metabolism	115654704	115658693	M.Prst
chr7	112300000	116400000	Ncf4	Metabolism	116167650	116190387	M.Prst
chr7	112300000	116400000	Csf2rb	Receptor	116237279	116272439	M.Liv
chr7	112300000	116400000	Tst	Binding protein	116346601	116355921	M.Kdn

chr7	112300000	116400000	Mpst	Metabolism	116359465	116364254	M.Kdn
chr8	45400000	49300000	Oaf	Development	46224861	46242832	M.Kdn
chr8	45400000	49300000	Pvrl1	Receptor	46739494	46798633	M.Prst
chr8	45400000	49300000	Rnf26	Protease	47093834	47095108	M.Prst
chr8	45400000	49300000	Tmem24	NA	47288755	47299021	M.Prst
chr8	45400000	49300000	Tmem24	NA	47288755	47299021	M.Liv
chr8	45400000	49300000	Dpagt1	Metabolism	47305087	47311488	M.Prst
chr8	45400000	49300000	Dpagt1	Metabolism	47305087	47311488	M.Kdn
chr8	45400000	49300000	Hmbs	Metabolism	47314235	47321604	M.Tst
chr8	45400000	49300000	Vps11	Transport	47324512	47339213	M.Prst
chr8	45400000	49300000	Hyou1	Protein binding	47346753	47358869	M.SV
chr8	45400000	49300000	Hyou1	Protein binding	47346753	47358869	M.Prst
chr8	45400000	49300000	---	NA	47651444	47651576	M.Kdn
chr8	45400000	49300000	Atp5l	Metabolism	47869992	47876824	M.Tst
chr8	45400000	49300000	Mpzl2	Extracellular matrix	47991858	48001888	M.Liv
chr8	45400000	49300000	ENSRNOT00000053557	NA	48018953	48019084	M.Prst
chr8	112800000	115850000	Slc38a3	Metabolism	112898377	112914473	M.Tst
chr8	112800000	115850000	Amigo3	Cytoskeleton	113321258	113322784	M.SV
chr8	112800000	115850000	Impdh2	Metabolism	113607025	113611615	M.Kdn
chr8	112800000	115850000	Ip6k2	Signaling	113836756	113861897	M.Liv
chr8	112800000	115850000	Slc26a6	Metabolism	113925291	113935357	M.Prst
chr8	112800000	115850000	Plxnb1	Signaling	114114234	114132960	M.Liv
chr8	112800000	115850000	Spink8	Protease	114190549	114202178	M.Tst
chr8	112800000	115850000	Nme6	Signaling	114205318	114212486	M.Tst
chr8	112800000	115850000	Map4	Cytoskeleton	114300908	114438008	M.Tst
chr8	112800000	115850000	Scap	Miscellaneous	114706464	114761344	M.Prst
chr8	112800000	115850000	Ptpn23	Signaling	114761478	114784605	M.Prst
chr8	112800000	115850000	Klhl18	Cytoskeleton	114802806	114861134	M.Kdn
chr8	112800000	115850000	Nbeal2	Signaling	115014718	115026066	M.Prst
chr8	112800000	115850000	Pthr1	NA	115099659	115120499	M.Liv
chr9	72250000	75550000	Aamp	Development	73606380	73611351	M.Prst
chr9	72250000	75550000	Aamp	Development	73606380	73611351	M.Kdn
chr9	72250000	75550000	Pnkd	Metabolism	73611608	73695976	M.Prst
chr9	72250000	75550000	Slc11a1	Transport	73714692	73725523	M.Prst
chr9	72250000	75550000	Rqcd1	Signaling	73843388	73870449	M.Prst
chr9	72250000	75550000	Rqcd1	Signaling	73843388	73870449	M.Hrt
chr9	72250000	75550000	Plcd4	Metabolism	73872916	73905127	M.Kdn
chr9	72250000	75550000	Stk36	Signaling	73951093	73976955	M.Tst
chr9	72250000	75550000	Stk36	Signaling	73951093	73976955	M.Kdn
chr9	72250000	75550000	ENSRNOT00000057365	NA	74207404	74213746	M.Tst
chr9	72250000	75550000	Des	Cytoskeleton	74637786	74645503	M.Kdn
chr9	72250000	75550000	Stk11ip	Signaling	74799499	74813957	M.Prst
chr10	38900000	41200000	Irf1	Transcription	39220592	39227698	M.Liv
chr10	38900000	41200000	LOC303140	Metabolism	39409212	39426730	M.Kdn
chr10	38900000	41200000	P4ha2	Metabolism	39522211	39550648	M.SV
chr10	38900000	41200000	P4ha2	Metabolism	39522211	39550648	M.Kdn
chr10	38900000	41200000	Cdc42se2	Development	40116093	40184995	M.Prst
chr10	38900000	41200000	Hint1	Signaling	40265633	40275627	M.Tst
chr10	38900000	41200000	Rpl13	Translation	40386057	40386798	M.Kdn
chr10	38900000	41200000	Slc36a1	Metabolism	40609213	40639096	M.Prst
chr10	38900000	41200000	Atox1	Metabolism	40858190	40873250	M.Kdn
chr10	38900000	41200000	ENSRNOT00000054033	NA	40892323	40892463	M.Prst
chr10	54750000	58150000	Slc25a35	Binding protein	55716718	55731778	M.Tst
chr10	54750000	58150000	Per1	Transcription	55858223	55869279	M.Liv
chr10	54750000	58150000	Per1	Transcription	55858223	55869279	M.Kdn
chr10	54750000	58150000	Per1	Transcription	55858223	55869279	M.Hrt

chr10	54750000	58150000	Tmem88	Miscellaneous	56191755	56192722	M.Kdn
chr10	54750000	58150000	Fxr2	Development	56450028	56470419	M.Prst
chr10	54750000	58150000	Cd68	Immune	56481701	56483580	M.SV
chr10	54750000	58150000	Tmem102	Miscellaneous	56624958	56636020	M.Prst
chr10	54750000	58150000	Plscr3	Binding protein	56687086	56691601	M.SV
chr10	54750000	58150000	Tnk1	Signaling	56692319	56701295	M.Prst
chr10	54750000	58150000	Pelp1	Transcription	57220322	57237359	M.Tst
chr10	54750000	58150000	Pelp1	Transcription	57220322	57237359	M.Prst
chr10	54750000	58150000	Pfn1	Cytoskeleton	57531658	57534366	M.Kdn
chr10	54750000	58150000	Rpain	Cytoskeleton	57874557	57881654	M.SV
chr10	62450000	64800000	Dph1	Miscellaneous	62486391	62492466	M.Prst
chr10	62450000	64800000	ENSRNOT00000041305	NA	62672302	62672591	M.Kdn
chr10	62450000	64800000	Prpf8	Translation	62808102	62830938	M.Tst
chr10	62450000	64800000	Slc43a2	Metabolism	62853990	62892164	M.Tst
chr10	62450000	64800000	Pitpna	Metabolism	62908505	62948857	M.Prst
chr10	62450000	64800000	Vps53	Proteolysis	63411061	63535551	M.Prst
chr10	62450000	64800000	Dbil5	Signaling	63571301	63572315	M.Tst
chr10	62450000	64800000	Pigs	Metabolism	64292136	64307281	M.Prst
chr10	62450000	64800000	ENSRNOT00000054438	NA	64334683	64334778	M.Kdn
chr10	62450000	64800000	Rab34	Signaling	64443111	64447076	M.SV
chr10	62450000	64800000	RGD1566149	NA	64481184	64482790	M.Tst
chr10	62450000	64800000	Tmem97	Miscellaneous	64652641	64661724	M.Hrt
chr10	86550000	91600000	BC099108	NA	86851151	86851437	M.Tst
chr10	86550000	91600000	Fbxl20	Transcription	86856617	86912521	M.Prst
chr10	86550000	91600000	Zpbp2	Development	87370697	87379602	M.SV
chr10	86550000	91600000	Zpbp2	Development	87370697	87379602	M.Liv
chr10	86550000	91600000	Rpl35a	Translation	87510023	87510441	M.Tst
chr10	86550000	91600000	Nr1d1	Transcription	87541260	87548186	M.SV
chr10	86550000	91600000	Rara	Signaling	87731006	87753265	M.Liv
chr10	86550000	91600000	Ccr7	Receptor	87924285	87936325	M.Kdn
chr10	86550000	91600000	Krt40	Cytoskeleton	88497766	88498065	M.Kdn
chr10	86550000	91600000	ENSRNOT00000045189	NA	88504916	88505215	M.Kdn
chr10	86550000	91600000	ENSRNOT00000048840	NA	88557017	88557409	M.Kdn
chr10	86550000	91600000	ENSRNOT00000048840	NA	88569311	88569703	M.Kdn
chr10	86550000	91600000	ENSRNOT00000048840	NA	88575672	88576064	M.Kdn
chr10	86550000	91600000	LOC680286	NA	88737031	88737753	M.Kdn
chr10	86550000	91600000	Krtap9-1	NA	88794615	88804818	M.Prst
chr10	86550000	91600000	Eif1	Translation	89255314	89257947	M.SV
chr10	86550000	91600000	Eif1	Translation	89255314	89257947	M.Prst
chr10	86550000	91600000	Jup	Cytoskeleton	89307938	89335556	M.Prst
chr10	86550000	91600000	Klhl11	Transcription	89407635	89417882	M.Hrt
chr10	86550000	91600000	Nkiras2	Signaling	89567507	89571663	M.Prst
chr10	86550000	91600000	Gcn5l2	NA	89642024	89649944	M.Prst
chr10	86550000	91600000	Hspb9	Signaling	89651116	89651870	M.Tst
chr10	86550000	91600000	Atp6v0a1	Transport	89948286	90072304	M.Prst
chr10	86550000	91600000	Becn1	Protease	90317953	90333329	M.Prst
chr10	86550000	91600000	Vat1	Metabolism	90483845	90491463	M.Kdn
chr10	86550000	91600000	Rnd2	Signaling	90493477	90497050	M.Tst
chr10	86550000	91600000	Arf4l	NA	90700494	90702029	M.Liv
chr10	86550000	91600000	Mpp2	Signaling	91126327	91153723	M.Prst
chr10	86550000	91600000	Nags	Metabolism	91211526	91215565	M.Prst
chr10	86550000	91600000	RGD1311493	NA	91368941	91374644	M.Prst
chr10	104150000	108000000	Ubn1	Transcription	10612475	10647858	M.Prst
chr10	104150000	108000000	N-pac	NA	10648071	10683534	M.Prst
chr10	104150000	108000000	Anks3	Transcription	10732109	10752778	M.Prst
chr10	104150000	108000000	Mgrn1	Protease	10756804	10805883	M.Prst

chr10	104150000	108000000	LOC363543	NA	10809552	10810469	M.Kdn
chr10	104150000	108000000	Gprc5c	Signaling	104753532	104753761	M.Prst
chr10	104150000	108000000	RGD1561778	Immune	105084291	105089251	M.Prst
chr10	104150000	108000000	RGD1561778	Immune	105084291	105089251	M.Liv
chr10	104150000	108000000	Cd300le	NA	105102015	105111669	M.Kdn
chr10	104150000	108000000	Slc9a3r1	Development	105237637	105254750	M.SV
chr10	104150000	108000000	Slc9a3r1	Development	105237637	105254750	M.Prst
chr10	104150000	108000000	Mrps7	Translation	105683751	105686890	M.Tst
chr10	104150000	108000000	Slc25a19	Binding protein	105694526	105707452	M.SV
chr10	104150000	108000000	Rps18	Translation	106078133	106078669	M.Tst
chr10	104150000	108000000	Itgb4	Signaling	106080448	106116634	M.Prst
chr10	104150000	108000000	Mrpl38	Binding protein	106238696	106245499	M.Kdn
chr10	104150000	108000000	RGD1311078	NA	106318430	106327931	M.Kdn
chr10	104150000	108000000	Ube2o	Protease	106648350	106694554	M.Prst
chr10	104150000	108000000	Cygb	Binding protein	106759885	106769651	M.SV
chr10	104150000	108000000	RGD1306284	NA	106948784	106953448	M.Prst
chr10	104150000	108000000	RGD1307394	NA	106957786	106971951	M.Prst
chr10	104150000	108000000	ENSRNOT00000069295	NA	107176182	107176365	M.Kdn
chr10	104150000	108000000	Sec14l1	Metabolism	107220888	107267940	M.Tst
chr10	104150000	108000000	Cant1	Signaling	108405355	108420225	M.Prst
chr12	15650000	17750000	Gpr146	Receptor	15755504	15756505	M.Kdn
chr12	15650000	17750000	Mir339	NA	15778323	15778418	M.Kdn
chr12	15650000	17750000	RGD1311660	NA	15878193	15895538	M.Kdn
chr12	15650000	17750000	Unc84a	NA	15899097	15931964	M.Prst
chr12	15650000	17750000	LOC288526	NA	16818259	16822099	M.Hrt
chr12	15650000	17750000	Asmtl	Epigenetics	16823122	16825697	M.Kdn
chr12	15650000	17750000	Rpl31	Translation	17453691	17454068	M.Tst
chr12	15650000	17750000	Cops6	Development	17598324	17606825	M.Prst
chr12	15650000	17750000	Taf6	Transcription	17620900	17629281	M.Prst
chr12	15650000	17750000	Mblac1	Metabolism and Transport	17636473	17637737	M.Kdn
chr12	21300000	24050000	Polr2j	Transcription	21658728	21663906	M.Tst
chr12	21300000	24050000	Por	Metabolism	22078629	22154339	M.Kdn
chr12	21300000	24050000	Rhbdd2	Protease	22198272	22208555	M.Prst
chr12	21300000	24050000	Rhbdd2	Protease	22198272	22208555	M.Kdn
chr12	21300000	24050000	Bcl7b	Transcription	22652182	22656139	M.SV
chr12	21300000	24050000	Dnajc30	Protein binding	22723862	22724380	M.Kdn
chr12	21300000	24050000	Abhd11	Metabolism	22777339	22780065	M.Prst
chr12	21300000	24050000	Wbscr27	Epigenetic	22822307	22835028	M.Kdn
chr12	21300000	24050000	Eif4h	Translation	23148332	23164980	M.Prst
chr12	21300000	24050000	Clip2	Cytoskeleton	23228309	23292105	M.Prst
chr12	21300000	24050000	---	NA	23731909	23732126	M.SV
chr12	31750000	34300000	Ubc	Translation	32333187	32337949	M.SV
chr12	31750000	34300000	Ubc	Translation	32333187	32337949	M.Prst
chr12	31750000	34300000	Zfoc1	NA	32871191	32872043	M.Tst
chr12	31750000	34300000	Ccdc92	Transcription	32919640	32923724	M.Hrt
chr12	31750000	34300000	Atp6v0a2	Transport	33057196	33119921	M.Prst
chr12	31750000	34300000	Eif2b1	Translation	33138323	33146597	M.Prst
chr12	31750000	34300000	Setd8-ps1	NA	33253255	33271667	M.Prst
chr12	31750000	34300000	Pitpnm2	Transport	33578103	33600689	M.Prst
chr12	31750000	34300000	Vps37b	Proteolysis	33684374	33713076	M.Tst
chr12	31750000	34300000	Hip1r	Cytoskeleton	33714261	33742792	M.Prst
chr12	31750000	34300000	Ccdc62	Transcription	33747236	33783801	M.Kdn
chr12	41000000	44700000	Gcn1l1	Signaling	42253353	42315249	M.SV
chr12	41000000	44700000	Pxn	Metabolism	42322188	42370508	M.Tst
chr12	41000000	44700000	Pxn	Metabolism	42322188	42370508	M.Prst
chr12	41000000	44700000	Rnf10	Miscellaneous	42609868	42643662	M.Prst

chr12	41000000	44700000	Pop5	Metabolism	42645162	42647081	M.Tst
chr12	41000000	44700000	Mlec	Signaling	42729870	42740346	M.Prst
chr12	41000000	44700000	Acads	Metabolism	42765265	42774529	M.Tst
chr12	41000000	44700000	Sppl3	Protease	42788815	42873133	M.Tst
chr12	41000000	44700000	Sppl3	Protease	42788815	42873133	M.Prst
chr12	41000000	44700000	RGD1311899	Miscellaneous	42945683	42951259	M.Kdn
chr12	41000000	44700000	Oasl2	Metabolism	42996518	43009338	M.Prst
chr12	41000000	44700000	Gltp	Metabolism	43193327	43215692	M.Kdn
chr12	41000000	44700000	Gltp	Metabolism	43193327	43215692	M.Hrt
chr12	41000000	44700000	Acacb	Metabolism	43388679	43493013	M.Prst
chr12	41000000	44700000	Sart3	Transcription	43893484	43920423	M.Prst
chr12	41000000	44700000	Ficd	Signaling	43924661	43927515	M.SV
chr12	41000000	44700000	Ficd	Signaling	43924661	43927515	M.Liv
chr13	85650000	89050000	---	NA	85798486	85798670	M.Hrt
chr13	85650000	89050000	ENSRNOT00000053447	NA	86791450	86791613	M.Tst
chr13	85650000	89050000	ENSRNOT00000053447	NA	86791450	86791613	M.Kdn
chr13	85650000	89050000	Fcgr2b	Immune	86809005	86823601	M.Prst
chr13	85650000	89050000	Tomm40b	NA	87109486	87112175	M.Tst
chr13	85650000	89050000	Fcer1g	Immune	87119755	87123821	M.Liv
chr13	85650000	89050000	Ppox	Metabolism	87170644	87174721	M.Liv
chr13	85650000	89050000	Usp21	Protease	87175893	87180031	M.Tst
chr13	85650000	89050000	F11r	Extracellular matrix	87369918	87393508	M.Prst
chr13	85650000	89050000	FQ230857	NA	87637815	87639122	M.Kdn
chr13	85650000	89050000	FQ230857	NA	87637815	87639122	M.Hrt
chr13	85650000	89050000	Cd84	Immune	87830393	87859987	M.Prst
chr13	85650000	89050000	Pea15a	Development	88187674	88197336	M.Kdn
chr13	85650000	89050000	Tagln2	Cytoskeleton	88505258	88512178	M.Liv
chr14	36100000	40050000	Agpat9	Metabolism	9998566	10050154	M.SV
chr14	36100000	40050000	RGD1310958	NA	37160631	37208711	M.Kdn
chr14	36100000	40050000	ENSRNOT00000002991	NA	37213283	37292691	M.Liv
chr14	36100000	40050000	ENSRNOT00000002991	NA	37213283	37292691	M.Hrt
chr14	36100000	40050000	Ociad1	Immune	37301310	37318693	M.Prst
chr14	36100000	40050000	RGD1311309	NA	37453020	37594206	M.Tst
chr14	36100000	40050000	Nfxl1	Transcription	38054861	38246847	M.SV
chr14	36100000	40050000	Nfxl1	Transcription	38054861	38246847	M.Hrt
chr14	36100000	40050000	Nfxl1	Transcription	38067476	38067654	M.SV
chr14	36100000	40050000	Nfxl1	Transcription	38067868	38068029	M.SV
chr14	36100000	40050000	Nfxl1	Transcription	38067868	38068029	M.Kdn
chr14	36100000	40050000	Nfxl1	Transcription	38068126	38068226	M.SV
chr14	36100000	40050000	Nfxl1	Transcription	38068776	38068890	M.SV
chr14	36100000	40050000	Nfxl1	Transcription	38070038	38070162	M.SV
chr14	36100000	40050000	Nfxl1	Transcription	38070806	38070928	M.Kdn
chr14	36100000	40050000	Nfxl1	Transcription	38070806	38070928	M.Hrt
chr14	36100000	40050000	Nfxl1	Transcription	38074519	38074609	M.SV
chr14	36100000	40050000	Nfxl1	Transcription	38074519	38074609	M.Kdn
chr14	36100000	40050000	Nfxl1	Transcription	38078341	38078461	M.SV
chr14	36100000	40050000	RGD1359201	NA	38078674	38095841	M.SV
chr14	82200000	85400000	Agpat9	Metabolism	9998566	10050154	M.SV
chr14	82200000	85400000	Tmem129	Miscellaneous	82761630	82777244	M.Prst
chr14	82200000	85400000	Maea	Receptors	83124774	83158198	M.Prst
chr14	82200000	85400000	Ywhah	Protein binding	83448395	83457804	M.Prst
chr14	82200000	85400000	Depdc5	Transcription	83487582	83622303	M.Tst
chr14	82200000	85400000	Depdc5	Transcription	83487582	83622303	M.Liv
chr14	82200000	85400000	Atp5e	NA	83615556	83615939	M.Tst
chr14	82200000	85400000	Atp5e	NA	83615556	83615939	M.Kdn
chr14	82200000	85400000	RGD1305469	NA	83631804	83647391	M.SV

chr14	82200000	85400000	RGD1560636	NA	83742995	83808033	M.SV
chr14	82200000	85400000	Selm	Metabolism	84157880	84160220	M.Kdn
chr14	82200000	85400000	Dusp18	Signaling	84555975	84560365	M.SV
chr14	82200000	85400000	Sf3a1	Translation	84786086	84804728	M.Tst
chr14	82200000	85400000	LOC685322	NA	85353539	85353912	M.Tst
chr15	31900000	34850000	Y09171	NA	32233140	32233187	M.Kdn
chr15	31900000	34850000	Lrp10	Receptor	32527394	32533554	M.Prst
chr15	31900000	34850000	RGD1565222	NA	32656916	32671931	M.Prst
chr15	31900000	34850000	Psmb5	Protease	32689865	32696834	M.Tst
chr15	31900000	34850000	Homez	Development	32948644	32965217	M.Liv
chr15	31900000	34850000	Homez	Development	32948644	32965217	M.Hrt
chr15	31900000	34850000	Zfhx2	NA	33175548	33175622	M.Prst
chr15	31900000	34850000	Thtpa	Metabolism	33201426	33205065	M.Kdn
chr15	31900000	34850000	Tinf2	Transcription	33826849	33832032	M.Prst
chr15	31900000	34850000	Rabggta	Metabolism	33861447	33868619	M.Prst
chr15	31900000	34850000	Rabggta	Metabolism	33861447	33868619	M.Kdn
chr15	31900000	34850000	Dhrs1	Metabolism	33900694	33907979	M.Tst
chr15	31900000	34850000	Mcpt2	Proteolysis	34287660	34288305	M.Prst
chr15	31900000	34850000	Mcpt2	Proteolysis	34287660	34288305	M.Kdn
chr15	31900000	34850000	Mcpt1	Proteolysis	34589939	34592578	M.Prst
chr16	17450000	21500000	Slc35e1	Metabolism	17769201	17779363	M.Prst
chr16	17450000	21500000	Use1	Golgi apparatus	18528294	18530994	M.Tst
chr16	17450000	21500000	Mrpl34	Translation	18636144	18636879	M.Kdn
chr16	17450000	21500000	Bst2	Signaling	18698540	18701768	M.Prst
chr16	17450000	21500000	Pik3r2	Signaling	19171101	19179650	M.Prst
chr16	17450000	21500000	Ifi30	Metabolism	19181236	19185449	M.Kdn
chr16	17450000	21500000	Rab3a	Signaling	19189765	19193874	M.Prst
chr16	17450000	21500000	Jund	Transcription	19239694	19241529	M.Prst
chr16	17450000	21500000	Ell	Transcription	19349937	19397249	M.Liv
chr16	17450000	21500000	Uba52	Proteolysis	19425409	19427555	M.Hrt
chr16	17450000	21500000	Rent1	NA	19576760	19579850	M.Prst
chr16	17450000	21500000	Slc25a42	Binding protein	19711607	19718584	M.Tst
chr16	17450000	21500000	RGD1308759	NA	19867130	19892848	M.Prst
chr16	17450000	21500000	Gatad2a	Transcription	19974633	20003490	M.Prst
chr16	17450000	21500000	Atp13a1	Metabolism and Transport	20100481	20117031	M.Prst
chr16	17450000	21500000	ENSRNOT00000034446	NA	20129174	20160654	M.Kdn
chr16	17450000	21500000	FQ211569	NA	20181460	20185669	M.Kdn
chr16	17450000	21500000	MGC72612	NA	20251590	20265573	M.Prst
chr16	17450000	21500000	MGC72612	NA	20251590	20265573	M.Kdn
chr16	17450000	21500000	ENSRNOT00000051889	NA	20346052	20347290	M.Tst
chr19	23600000	26800000	RGD1564739	NA	24278128	24279626	M.Hrt
chr19	23600000	26800000	Tnpo2	Binding protein	24757439	24775871	M.Prst
chr19	23600000	26800000	Hook2	Transport	24808551	24819397	M.SV
chr19	23600000	26800000	Prdx2	Metabolism	24836198	24841414	M.Tst
chr19	23600000	26800000	Prdx2	Metabolism	24836198	24841414	M.Kdn
chr19	23600000	26800000	Gcdh	Metabolism and Transport	24919668	24926084	M.SV
chr19	23600000	26800000	Rad23a	DNA repair	24971070	24976904	M.Tst
chr19	23600000	26800000	Gadd45gip1	Epigenetics	24977273	24979410	M.Kdn
chr19	23600000	26800000	Nfix	Transcription	25018391	25111495	M.Prst
chr19	23600000	26800000	Mri1	Translation	25591115	25597107	M.Kdn
chr19	23600000	26800000	Cd97	Receptor	26089268	26108999	M.Liv
chr19	23600000	26800000	Dnajb1	Protein binding	26213753	26217480	M.Hrt
chr19	23600000	26800000	Gpsn2	NA	26232602	26259289	M.Prst
chr19	23600000	26800000	RGD1308221	NA	26572817	26672476	M.Prst
chr19	34950000	37650000	Tmem208	Miscellaneous	35141501	35143925	M.Tst
chr19	34950000	37650000	Pskh1	Signaling	35740175	35773790	M.Prst

chr19	34950000	37650000	Pskh1	Signaling	35740175	35773790	M.Kdn
chr19	34950000	37650000	Ddx28	Transcription	35856843	35858465	M.Prst
chr19	34950000	37650000	Nfatc3	Transcription	35907874	35979801	M.Tst
chr19	34950000	37650000	Rps12	Translation	36293808	36294342	M.Kdn
chr19	34950000	37650000	Cirh1a	Development	36742206	36770285	M.Tst
chr19	34950000	37650000	Sntb2	Cytoskeleton	36781979	36866566	M.Hrt
chr19	34950000	37650000	LOC690214	NA	36902778	36904111	M.Kdn
chr19	34950000	37650000	Cog8	Metabolism	36904343	36914964	M.Prst
chr19	34950000	37650000	Nqo1	Metabolism	37251087	37266040	M.Kdn
chr20	2000000	8050000	Tubb5	Cytoskeleton	3060237	3064785	M.Kdn
chr20	2000000	8050000	Bat1	NA	3611128	3623527	M.Tst
chr20	2000000	8050000	ENSRNOT00000052790	NA	3622576	3622654	M.Tst
chr20	2000000	8050000	Bat2	NA	3724278	3737617	M.Prst
chr20	2000000	8050000	ENSRNOT00000052797	NA	3962124	3962187	M.Tst
chr20	2000000	8050000	Neu1	Metabolism	3999328	4003675	M.Prst
chr20	2000000	8050000	Neu1	Metabolism	3999328	4003675	M.Kdn
chr20	2000000	8050000	Atf6b	Transcription	4192185	4200108	M.Prst
chr20	2000000	8050000	Agpat1	Metabolism	4237798	4245723	M.Tst
chr20	2000000	8050000	Psmb8	Protease	4786264	4789596	M.Prst
chr20	2000000	8050000	Hla-dma	NA	4843458	4846844	M.Kdn
chr20	2000000	8050000	ENSRNOT00000036804	NA	4909280	4917172	M.Prst
chr20	2000000	8050000	Rps18	Translation	5083626	5087304	M.Tst
chr20	2000000	8050000	Tapbp	Immune	5096644	5117749	M.Kdn
chr20	2000000	8050000	Phf1	Epigenetics	5170050	5175029	M.Prst
chr20	2000000	8050000	Itpr3	Transport	5292430	5357502	M.Prst
chr20	2000000	8050000	RGD1306917	NA	5358004	5371506	M.Tst
chr20	2000000	8050000	Nudt3	Signaling	5789033	5841633	M.Kdn
chr20	2000000	8050000	Spdef	Transcription	5928300	5934265	M.SV
chr20	2000000	8050000	RGD1307264	NA	6066778	6114705	M.Prst
chr20	2000000	8050000	Taf11	Transcription	6113417	6119513	M.Hrt
chr20	2000000	8050000	Znf76	NA	6417556	6446496	M.Prst
chr20	2000000	8050000	Mapk13	Signaling	7055313	7064405	M.Prst
chr20	2000000	8050000	Brpf3	Transcription	7090904	7121514	M.Tst
chr20	2000000	8050000	Cdkn1a	Cell cycle	7376325	7386778	M.Prst
chr20	2000000	8050000	U15425	NA	7657695	7658977	M.Prst
chr20	2000000	8050000	Tbc1d22b	Signaling	7880190	7921453	M.Prst
chr20	2000000	8050000	Rnf8	Proteolysis	7929796	7954697	M.Tst
chr20	2000000	8050000	Ftsjd2	NA	7957560	8014387	M.Prst
chr20	2000000	8050000	Tbrg1	Growth factor	30830696	30832163	M.Prst
chr20	2000000	8050000	Dcbld1	Development	30945776	31037959	M.Prst
chr20	2000000	8050000	Pln	Metabolism	32000371	32008559	M.Prst
chr20	2000000	8050000	---	NA	33057448	33058521	M.Tst
chr20	2000000	8050000	LOC100359595	NA	34820149	35021979	M.Kdn
chr20	2000000	8050000	GENSCAN00000021698	NA	36088033	36089088	M.Kdn
chr20	2000000	8050000	Tspyl4	Protein binding	38639281	38641288	M.Liv
chr20	2000000	8050000	ENSRNOT00000052893	NA	39307650	39307790	M.Hrt
chr20	2000000	8050000	Rpl13a	Translation	41125183	41125850	M.Tst
chr20	2000000	8050000	Fyn	Signaling	43501853	43695567	M.Liv
chr20	2000000	8050000	Slc16a10	Transport	44211646	44325332	M.Prst
chr20	2000000	8050000	Bxdc1	NA	44388616	44410101	M.Liv
chr20	2000000	8050000	J01884	NA	44425033	44425245	M.Tst
chr20	2000000	8050000	Zbtb24	Transcription	45408881	45425056	M.Prst
chr20	2000000	8050000	Foxo3	Transcription	46171683	46261910	M.Prst
chr20	2000000	8050000	Sec63	Protein binding	46669289	46734882	M.Prst
chr20	2000000	8050000	Fxc1	NA	47476320	47476986	M.Prst
chr20	2000000	8050000	Fxc1	NA	47476320	47476986	M.Kdn

chr20	2000000	8050000	Rtn4ip1	Metabolism	47818478	47857755	M.Prst
chr20	2000000	8050000	Aim1	NA	47862449	47919390	M.Prst
chr20	2000000	8050000	Aim1	NA	47919270	47921443	M.Prst
