# Supplementary material for: Genomic Clustering of differential DNA methylated regions (epimutations) associated with the epigenetic transgenerational inheritance of disease and phenotypic variation
Source: BMC Genomics. 2016 Jun 1;17:418. doi: 10.1186/s12864-016-2748-5 (PMC4888261; doi:10.1186/s12864-016-2748-5)
Supplement: Additional file 3: Table S2. — Female gene clusters. Gene cluster sites with start-end location of each cluster site, genes in each cluster and classification, and gene start and stop, and types of the tissue for the Female Tissue Array dataset. (PDF 59 kb) [file 12864_2016_2748_MOESM3_ESM.pdf]

Supplemental Table S2 Female Tissue Gene Clusters

Cluster Chromosome	ClusterStart	ClusterEnd	Gene.Name	Gene Classification	Gene cSTART	Gene cSTOP	Tissue Type
chr1	81050000	84100000	Pex3		8316113	8358052	Fm.Liv
chr1	81050000	84100000	Sdccag1		82120277	82122428	Fm.Hrt
chr1	81050000	84100000	Egln2		82233239	82241258	Fm.Uter
chr1	81050000	84100000	Ltbp4		82385958	82418177	Fm.Uter
chr1	81050000	84100000	ENSRNOT00000057163		82734707	82736642	Fm.Ov
chr1	81050000	84100000	LOC687333		83003444	83016695	Fm.Hrt
chr1	93900000	96700000	LOC499144		93986011	93992823	Fm.Liv
chr1	93900000	96700000	RGD1309326		94714136	94720015	Fm.Hrt
chr1	93900000	96700000	Tbc1d17		95313610	95321624	Fm.Kdn
chr1	93900000	96700000	Prr12		95501946	95525617	Fm.Uter
chr1	93900000	96700000	Snrp70		95847853	95868392	Fm.Hrt
chr1	93900000	96700000	Snrp70		95847853	95868392	Fm.Kdn
chr1	109350000	113350000	ENSRNOT00000053327		110871873	110871950	Fm.Uter
chr1	109350000	113350000	ENSRNOT00000053327		110873709	110873786	Fm.Uter
chr1	109350000	113350000	ENSRNOT00000053882		111288045	111288137	Fm.Ov
chr1	109350000	113350000	ENSRNOT00000054014		111338314	111338406	Fm.Ov
chr1	109350000	113350000	ENSRNOT00000052564		111349177	111349269	Fm.Ov
chr1	109350000	113350000	ENSRNOT00000053882		111360802	111360894	Fm.Ov
chr1	109350000	113350000	ENSRNOT00000053882		111371982	111372074	Fm.Ov
chr1	109350000	113350000	ENSRNOT00000053882		111374556	111374648	Fm.Ov
chr1	109350000	113350000	ENSRNOT00000053529		111377258	111377350	Fm.Ov
chr1	109350000	113350000	ENSRNOT00000053882		111386349	111386441	Fm.Ov
chr1	109350000	113350000	ENSRNOT00000053882		111388918	111389010	Fm.Ov
chr1	109350000	113350000	ENSRNOT00000053882		111391497	111391589	Fm.Ov
chr1	109350000	113350000	ENSRNOT00000053882		111399841	111399933	Fm.Ov
chr1	109350000	113350000	ENSRNOT00000053893		111454267	111454359	Fm.Ov
chr1	109350000	113350000	ENSRNOT00000053882		111456841	111456933	Fm.Ov
chr1	109350000	113350000	ENSRNOT00000053882		111467045	111467137	Fm.Ov
chr1	159650000	163550000	Hbb		161578261	161579458	Fm.Ov
chr1	159650000	163550000	Hbb		161578261	161579458	Fm.Uter
chr1	159650000	163550000	Hbb		161584858	161620198	Fm.Ov
chr1	159650000	163550000	Hbb		161584858	161620198	Fm.Uter
chr1	159650000	163550000	MGC72973		161590658	161598127	Fm.Ov
chr1	159650000	163550000	MGC72973		161590658	161598127	Fm.Uter
chr1	159650000	163550000	Rrp8		163477026	163481051	Fm.Ov
chr1	185850000	188000000	Ccdc95		186030833	186041424	Fm.Uter
chr1	185850000	188000000	Znf688		186684555	186687183	Fm.Uter
chr1	185850000	188000000	ENSRNOT00000053325		186812653	186812786	Fm.Liv
chr1	185850000	188000000	Tgfb1i1		187504568	187511512	Fm.Uter
chr1	185850000	188000000	Bag3		187780988	187804835	Fm.Uter
chr1	206400000	210350000	Drap1		208067901	208070604	Fm.Ov
chr1	206400000	210350000	Ccdc85b		208099948	208100579	Fm.Uter
chr1	206400000	210350000	Rela		208262669	208273148	Fm.Uter
chr1	206400000	210350000	Mtvr2		208355217	208356772	Fm.Ov
chr1	206400000	210350000	Ltbp3		208366405	208385177	Fm.Uter
chr1	206400000	210350000	BC090353		208428092	208428255	Fm.Hrt
chr1	206400000	210350000	Slc25a45		208513653	208519990	Fm.Liv
chr1	206400000	210350000	Mrpl49		208691822	208694923	Fm.Ov
chr1	206400000	210350000	LOC100361915		208705280	208712079	Fm.Uter
chr1	206400000	210350000	RGD1560108		210107906	210108290	Fm.Hrt
chr2	188800000	192900000	Lingo4		189345199	189369074	Fm.Uter
chr2	188800000	192900000	Oaz3		189408570	189417752	Fm.Ov
chr2	188800000	192900000	LOC100363361		189907124	189915547	Fm.Kdn

chr2	188800000	192900000	Car14	190683387	190690466	Fm.Ov
chr2	188800000	192900000	Plekho1	190787503	190796057	Fm.Uter
chr2	188800000	192900000	Sf3b4	190976645	190981640	Fm.Uter
chr2	188800000	192900000	Sv2a	190985435	191000859	Fm.Kdn
chr2	188800000	192900000	Rbm8	191429809	191432185	Fm.Uter
chr2	188800000	192900000	Gpr89	191676215	191700484	Fm.Hrt
chr2	188800000	192900000	FQ217306	192534080	192534354	Fm.Kdn
chr3	7200000	9800000	ENSRNOT00000052447	7826086	7826248	Fm.Ov
chr3	7200000	9800000	Setx	8099542	8156463	Fm.Hrt
chr3	7200000	9800000	Setx	8099542	8156463	Fm.Uter
chr3	7200000	9800000	Odf2	8877009	8921175	Fm.Ov
chr3	7200000	9800000	LOC499770	9187007	9194243	Fm.Kdn
chr3	7200000	9800000	Cry2	76802782	76830902	Fm.Uter
chr3	7200000	9800000	Hsd17b12	78436634	78559724	Fm.Uter
chr3	7200000	9800000	Ttc17	78706151	78815348	Fm.Hrt
chr3	7200000	9800000	ENSRNOT00000056863	88325556	88433892	Fm.Liv
chr3	7200000	9800000	ENSRNOT00000056831	89733136	89815718	Fm.Uter
chr3	7200000	9800000	Lin7c	95327942	95335254	Fm.Ov
chr3	107400000	111200000	Ndufb4	108822485	108823579	Fm.Ov
chr3	107400000	111200000	RGD1562529	108839136	108905119	Fm.Ov
chr3	107400000	111200000	Slc28a2	109203979	109204805	Fm.Hrt
chr3	107400000	111200000	Slc28a2	109203979	109204805	Fm.Liv
chr3	107400000	111200000	Slc28a2	109350531	109372027	Fm.Hrt
chr3	107400000	111200000	Slc28a2	109350531	109372027	Fm.Liv
chr3	107400000	111200000	MGC105649	109610054	109613454	Fm.Ov
chr3	112800000	116500000	Ptges2	11521725	11528396	Fm.Ov
chr3	112800000	116500000	Trpm7	114311685	114410273	Fm.Hrt
chr3	112800000	116500000	Ap4e1	114550196	114615095	Fm.Hrt
chr3	112800000	116500000	Ciao1	114740676	114746203	Fm.Ov
chr3	112800000	116500000	ENSRNOT00000055921	114767633	114767988	Fm.Hrt
chr3	112800000	116500000	ENSRNOT00000055921	114767633	114767988	Fm.Ov
chr3	112800000	116500000	Anapc1	116214677	116297953	Fm.Uter
chr4	26200000	30000000	ENSRNOT00000062069	2911511	2914338	Fm.Ov
chr4	26200000	30000000	Cyp51	26752542	26771131	Fm.Hrt
chr4	26200000	30000000	Samd9l	28016232	28016960	Fm.Kdn
chr4	26200000	30000000	Samd9l	28086298	28087026	Fm.Kdn
chr4	26200000	30000000	RGD1561472	28142515	28143240	Fm.Kdn
chr4	26200000	30000000	Samd9l	28180812	28185536	Fm.Kdn
chr4	26200000	30000000	Gng11	28847907	28853349	Fm.Ov
chr4	26200000	30000000	LOC500034	39063882	39128085	Fm.Ov
chr4	165300000	167300000	MGC94282	165378057	165383377	Fm.Hrt
chr4	165300000	167300000	Pzp	165505085	165551149	Fm.Uter
chr4	165300000	167300000	Klrb1c	166170376	166180934	Fm.Ov
chr4	165300000	167300000	Klri1	167017909	167056435	Fm.Ov
chr4	165300000	167300000	LOC690045	167274060	167298114	Fm.Liv
chr5	59900000	63150000	Tpm2	59994101	60003261	Fm.Kdn
chr5	59900000	63150000	Gba2	60045397	60057166	Fm.Hrt
chr5	59900000	63150000	Zcchc7	61167557	61413408	Fm.Hrt
chr5	59900000	63150000	Zcchc7	61472581	61477933	Fm.Hrt
chr5	59900000	63150000	Exosc3	61878632	61883826	Fm.Ov
chr5	59900000	63150000	---	62428918	62429008	Fm.Liv
chr5	59900000	63150000	Ncbp1	62691068	62723436	Fm.Hrt
chr5	142100000	144700000	Macf1	142659764	142986926	Fm.Hrt
chr5	142100000	144700000	LOC100366213	142717134	142719930	Fm.Hrt
chr5	142100000	144700000	ENSRNOT00000052635	142982420	142982524	Fm.Ov
chr5	142100000	144700000	Fhl3	144026350	144033665	Fm.Uter
chr5	142100000	144700000	Sf3a3	144040061	144059692	Fm.Uter

chr5	142100000	144700000	Mtf1	144137786	144177518	Fm.Ov
chr5	151750000	155500000	Smpdl3b	151982240	152124168	Fm.Hrt
chr5	151750000	155500000	Sh3bgrl3	152877349	152878653	Fm.Liv
chr5	151750000	155500000	Tmem57	153568577	153636412	Fm.Hrt
chr5	151750000	155500000	Tmem57	153568577	153636412	Fm.Ov
chr5	151750000	155500000	RGD1359529	153698998	153702612	Fm.Hrt
chr5	151750000	155500000	RGD1359529	153698998	153702612	Fm.Kdn
chr5	151750000	155500000	LOC689826	154164958	154166195	Fm.Uter
chr5	151750000	155500000	Fusip1	154637426	154648795	Fm.Hrt
chr6	125350000	128050000	Tc2n	125871936	125975388	Fm.Kdn
chr6	125350000	128050000	Trip11	126119081	126188777	Fm.Ov
chr6	125350000	128050000	Atxn3	126196043	126229844	Fm.Hrt
chr6	125350000	128050000	Cpsf2	126248830	126272054	Fm.Ov
chr6	125350000	128050000	ENSRNOT00000011530	127304349	127305516	Fm.Liv
chr6	125350000	128050000	Asb2	127609500	127645586	Fm.Ov
chr7	48350000	51900000	Nav3	48670216	48741044	Fm.Hrt
chr7	48350000	51900000	Nav3	48861714	48861956	Fm.Hrt
chr7	48350000	51900000	---	49931964	49932331	Fm.Ov
chr7	48350000	51900000	Zdhhc17	49933342	49995860	Fm.Hrt
chr7	48350000	51900000	RGD1561474	50214458	50313065	Fm.Hrt
chr7	48350000	51900000	Bbs10	50317899	50320409	Fm.Hrt
chr7	48350000	51900000	Phlda1	50541012	50542775	Fm.Uter
chr7	118800000	122650000	Tnrc6b	118993677	119201749	Fm.Hrt
chr7	118800000	122650000	ENSRNOT00000039879	119445434	119445806	Fm.Hrt
chr7	118800000	122650000	Cyp2d4v1	120741304	120752289	Fm.Uter
chr7	118800000	122650000	Cyp2d5	120760416	120764982	Fm.Hrt
chr7	118800000	122650000	Cyp2d5	120760416	120764982	Fm.Uter
chr7	118800000	122650000	Rrp7a	121146045	121153416	Fm.Ov
chr7	118800000	122650000	Ttll12	121600193	121621050	Fm.Ov
chr10	13600000	17550000	Axin1	15409373	15462726	Fm.Hrt
chr10	13600000	17550000	Luc7l	15520577	15550006	Fm.Kdn
chr10	13600000	17550000	Hba-a2	15554909	15571773	Fm.Ov
chr10	13600000	17550000	Hba-a2	15554909	15571773	Fm.Uter
chr10	13600000	17550000	LOC287167	15558732	15559543	Fm.Kdn
chr10	13600000	17550000	Hba-a2	15584359	15585215	Fm.Ov
chr10	13600000	17550000	Hba-a2	15584359	15585215	Fm.Uter
chr10	13600000	17550000	RGD1310922	15667569	15671373	Fm.Ov
chr10	55700000	58500000	Zbtb4	56579482	56602525	Fm.Uter
chr10	55700000	58500000	Slc2a4	56786705	56792254	Fm.Hrt
chr10	55700000	58500000	Rnasek	57077240	57078994	Fm.Uter
chr10	55700000	58500000	Alox15	57185939	57194401	Fm.Kdn
chr10	55700000	58500000	Zfp3	57664745	57666280	Fm.Liv
chr12	19650000	21650000	Agfg2	19715577	19828867	Fm.Uter
chr12	19650000	21650000	Mepce	19848918	19853448	Fm.Ov
chr12	19650000	21650000	Ap1s1	20908378	20918225	Fm.Ov
chr12	19650000	21650000	Serpine1	20931996	20942374	Fm.Uter
chr12	19650000	21650000	Prkrip1	21586197	21604474	Fm.Hrt
chr13	85750000	89600000	---	85798486	85798670	Fm.Ov
chr13	85750000	89600000	Uap1	85953105	85987210	Fm.Ov
chr13	85750000	89600000	Pcp4l1	87059477	87083054	Fm.Hrt
chr13	85750000	89600000	Apoa2	87114734	87116372	Fm.Liv
chr13	85750000	89600000	Cd48	87684509	87708091	Fm.Kdn
chr13	85750000	89600000	Atp1a2	88258993	88283933	Fm.Kdn
chr13	85750000	89600000	Kcnj10	88341102	88370591	Fm.Kdn
chr13	85750000	89600000	Pigm	88377212	88380909	Fm.Ov
chr13	85750000	89600000	Slamf9	88480220	88482958	Fm.Kdn
chr15	3450000	7300000	Spetex-2F	4719340	4721663	Fm.Ov

chr15	3450000	7300000	Spetex-2A	4738084	4740379	Fm.Ov
chr15	3450000	7300000	Spetex-2A	4982394	4985966	Fm.Ov
chr15	3450000	7300000	Spetex-2C	5381867	5385012	Fm.Ov
chr15	3450000	7300000	Spetex-2A	5435691	5439580	Fm.Ov
chr15	3450000	7300000	Spetex-2G	5494404	5598172	Fm.Ov
chr15	3450000	7300000	Spetex-2H	5547299	5550592	Fm.Ov
chr15	3450000	7300000	Spetex-2D	5922760	5925028	Fm.Ov
chr15	3450000	7300000	RGD1306353	35791554	35805587	Fm.Hrt
chr15	3450000	7300000	Zmym2	35921575	35978241	Fm.Hrt
chr15	3450000	7300000	Gjb2	36153526	36159490	Fm.Uter
chr15	3450000	7300000	Xpo4	36615866	36702294	Fm.Uter
chr15	3450000	7300000	ENSRNOT00000052659	38272646	38272741	Fm.Hrt
chr15	3450000	7300000	Nupl1	39301525	39350088	Fm.Hrt
chr15	3450000	7300000	Rnaseh2b	41554754	41597638	Fm.Ov
chr15	3450000	7300000	Ints6	41925924	42030867	Fm.Hrt
chr15	3450000	7300000	Zfp395	44796164	44817523	Fm.Uter
chr15	3450000	7300000	P2ry5	53875463	53877272	Fm.Kdn
chr15	3450000	7300000	Lcp1	56034457	56091880	Fm.Kdn
chr16	17300000	20300000	Tmem38a	17668988	17684448	Fm.Kdn
chr16	17300000	20300000	Cnot7	18385449	18386236	Fm.Kdn
chr16	17300000	20300000	Slc27a1	18769909	18786990	Fm.Uter
chr16	17300000	20300000	Ccdc124	19075014	19080086	Fm.Uter
chr16	17300000	20300000	Pgpep1	19276650	19285848	Fm.Liv
chr16	17300000	20300000	Ell	19349937	19397249	Fm.Uter
chr19	24550000	26950000	Fam96b	262448	264118	Fm.Ov
chr19	24550000	26950000	Nfix	25018391	25111495	Fm.Uter
chr19	24550000	26950000	Nacc1	25131751	25148664	Fm.Ov
chr19	24550000	26950000	Ier2	25159276	25160800	Fm.Hrt
chr19	24550000	26950000	Zswim4	25611348	25636716	Fm.Uter
chr19	24550000	26950000	Elmod2	26506221	26520608	Fm.Hrt
chr20	2750000	5850000	Ppp1r10	2970680	2984397	Fm.Ov
chr20	2750000	5850000	ENSRNOT00000052790	3622576	3622654	Fm.Kdn
chr20	2750000	5850000	Rpl13-ps1	3908012	3908634	Fm.Ov
chr20	2750000	5850000	Neu1	3999328	4003675	Fm.Uter
chr20	2750000	5850000	RT1-Bb	4730559	4737433	Fm.Kdn
chr20	2750000	5850000	Zbtb22	5118604	5120514	Fm.Uter
chr20	2750000	5850000	ENSRNOT00000059561	5148911	5149490	Fm.Hrt
chr20	2750000	5850000	Csrp2	29590539	29591118	Fm.Ov
chr20	2750000	5850000	Ddx21	29838671	29860514	Fm.Ov
chr20	2750000	5850000	Zufsp	30096106	30126201	Fm.Ov
chr20	2750000	5850000	---	33057448	33058521	Fm.Kdn
chr20	2750000	5850000	Ranbp2	37272405	37322765	Fm.Hrt
chr20	2750000	5850000	RGD1560095	38442095	38514255	Fm.Hrt
chr20	2750000	5850000	Lama4	43078138	43260577	Fm.Ov
chr20	2750000	5850000	LOC100188933	43266881	43268129	Fm.Ov
chr20	2750000	5850000	Fyn	43501853	43695567	Fm.Uter
chr20	2750000	5850000	Traf3ip2	43747622	43792258	Fm.Ov
chr20	2750000	5850000	Qrsl1	47679218	47818351	Fm.Uter
chr20	2750000	5850000	Ascc3	54600906	54868857	Fm.Hrt
chrX	39000000	42250000	Gnl3l	40301229	40331804	Fm.Ov
chrX	39000000	42250000	---	40730725	40730779	Fm.Ov
chrX	39000000	42250000	RGD1561065	40871138	40967234	Fm.Hrt
chrX	39000000	42250000	RGD1561065	40871138	40967234	Fm.Kdn
chrX	39000000	42250000	Smc1a	41503393	41547830	Fm.Ov
