# Supplementary material for: Genomic Clustering of differential DNA methylated regions (epimutations) associated with the epigenetic transgenerational inheritance of disease and phenotypic variation
Source: BMC Genomics. 2016 Jun 1;17:418. doi: 10.1186/s12864-016-2748-5 (PMC4888261; doi:10.1186/s12864-016-2748-5)
Supplement: Additional file 4: Table S3. — Sertoli DMR clusters. DMR clusters with start-end, statistical significance for each DMR with start and stop information for the Sertoli Cell dataset. (PDF 35 kb) [file 12864_2016_2748_MOESM4_ESM.pdf]

Supplemental Table S3 **Sertoli Cell DMR Cluster**

Cluster Chromosome	ClusterStart	ClusterEnd	DMR min P Value	DMR cSTART	DMR cSTOP
chr1	39150000	42350000	0.0445397	40399144	40400567
chr1	39150000	42350000	0.0143362	41054707	41092501
chr1	39150000	42350000	0.00472085	41097174	41104031
chr1	62450000	65850000	0.0306023	62798103	62801085
chr1	62450000	65850000	0.00000485	63868861	63885057
chr1	62450000	65850000	0.0400063	64400805	64401741
chr1	62450000	65850000	0.0347312	65242090	65247202
chr1	206250000	210350000	0.014464	206708922	206714409
chr1	206250000	210350000	0.0304948	207661537	207670801
chr1	206250000	210350000	0.00454151	208232325	208233267
chr1	206250000	210350000	0.0138794	208428092	208428255
chr1	206250000	210350000	0.00872725	208699058	208700411
chr1	206250000	210350000	0.0107973	209583414	209586571
chr2	188900000	191150000	0.0495313	189240524	189242900
chr2	188900000	191150000	0.0108908	189778476	189781169
chr2	188900000	191150000	0.0175331	190799602	190859954
chr3	3150000	7800000	0.0452556	3435393	3436406
chr3	3150000	7800000	0.0443675	4390427	4419424
chr3	3150000	7800000	0.0356916	5120291	5121217
chr3	3150000	7800000	0.038426	5809368	5809449
chr3	3150000	7800000	0.0300528	5810027	5810097
chr3	3150000	7800000	0.0369372	5817198	5830060
chr3	168850000	171000000	0.0180214	169089412	169099834
chr3	168850000	171000000	0.0382939	169712268	169731866
chr3	168850000	171000000	0.047346	170831286	170839091
chr5	77500000	81400000	0.0126496	79294671	79367278
chr5	77500000	81400000	0.00674035	79442642	79461272
chr5	77500000	81400000	0.0198293	79471099	79492969
chr5	77500000	81400000	0.0475767	80515699	80521861
chr5	134600000	138150000	0.00596212	136205260	136234065
chr5	134600000	138150000	0.046184	136310964	136360649
chr5	134600000	138150000	0.0184863	136497494	136566473
chr6	102050000	105600000	0.0370008	103693040	103694836
chr6	102050000	105600000	0.0263825	103727927	103729726
chr6	102050000	105600000	0.00289872	104036871	104041029
chr7	7950000	12350000	0.0148612	9028891	9030536
chr7	7950000	12350000	0.0416807	9510066	9532434
chr7	7950000	12350000	0.00651632	9936617	9939119
chr7	7950000	12350000	0.0329345	10367357	10369607
chr7	7950000	12350000	0.0329578	10685977	10705327
chr7	7950000	12350000	0.00827683	10976438	10991564
chr7	116000000	119850000	0.0422961	117266784	117307172
chr7	116000000	119850000	0.0310916	117903217	117908964
chr7	116000000	119850000	0.0277075	117934208	117969854
chr7	116000000	119850000	0.0322455	119794976	119805104
chr8	73000000	75750000	0.0288506	73034563	73818366
chr8	73000000	75750000	0.00973934	73836837	73876511

chr8	73000000	75750000	0.00487821	74909359	74983325
chr8	73000000	75750000	0.00489224	75202511	75289572
chr9	72550000	75700000	0.0153927	73780203	73831168
chr9	72550000	75700000	0.00345848	73951093	73976955
chr9	72550000	75700000	0.0207753	74547427	74548521
chr10	12150000	15050000	0.0337017	13051312	13084236
chr10	12150000	15050000	0.0435974	13722377	13724292
chr10	12150000	15050000	0.0224177	14103353	14117608
chr10	45700000	48600000	0.03697	46614082	46662673
chr10	45700000	48600000	0.0427018	47649198	47654653
chr10	45700000	48600000	0.00156688	47665622	47673226
chr10	53800000	59250000	0.0480638	55339250	55349087
chr10	53800000	59250000	0.0332359	55680917	55686475
chr10	53800000	59250000	0.0480317	55768368	55788834
chr10	53800000	59250000	0.0424029	55825991	55827700
chr10	53800000	59250000	0.0106524	56208345	56369356
chr10	53800000	59250000	0.0281562	56485162	56485304
chr10	53800000	59250000	0.0347788	56760289	56765325
chr10	53800000	59250000	0.0428298	56780470	56786053
chr10	53800000	59250000	0.0219766	57293752	57295539
chr10	53800000	59250000	0.0168829	57370395	57372706
chr10	53800000	59250000	0.0103073	57874557	57881654
chr10	88500000	92250000	0.0413218	90137813	90144403
chr10	88500000	92250000	0.0169739	90306766	90307737
chr10	88500000	92250000	0.00843692	90469785	90473512
chr10	88500000	92250000	0.034524	91581206	91581393
chr12	32400000	36300000	0.0406032	33977383	33997328
chr12	32400000	36300000	0.0199966	34196347	34209636
chr12	32400000	36300000	0.0449493	34382419	34393577
chr12	32400000	36300000	0.0367817	34412943	34463189
chr12	32400000	36300000	0.0225058	35412463	35443838
chr14	8150000	11050000	0.047394	9072989	9135391
chr14	8150000	11050000	0.0276051	10096519	10102420
chr14	8150000	11050000	0.0138289	10102482	10135653
chr14	81900000	85550000	0.00780089	82746460	82760864
chr14	81900000	85550000	0.0366503	83615556	83615939
chr14	81900000	85550000	0.0133893	83855894	83871922
chr14	81900000	85550000	0.0457239	85353539	85353912
chr16	30450000	33850000	0.0218235	31943480	31957555
chr16	30450000	33850000	0.00879214	32200642	32200814
chr16	30450000	33850000	0.0355941	32317666	32435983
chr17	48150000	52050000	0.00729179	48513674	48514066
chr17	48150000	52050000	0.0173306	48989449	48990680
chr17	48150000	52050000	0.0384544	50119480	50120573
chr17	48150000	52050000	0.0174517	50350988	50352473
chr17	48150000	52050000	0.0281426	50788000	50809123
chr19	22950000	26800000	0.0240019	24781386	24784715
chr19	22950000	26800000	0.0340153	24831536	24833320
chr19	22950000	26800000	0.0165459	24925342	24943271
chr19	22950000	26800000	0.0334597	25867705	25882323

chr19	38500000	42400000	0.0143947	40412611	40412788
chr19	38500000	42400000	0.0345194	40472657	40472756
chr19	38500000	42400000	0.0392472	40490890	40491177
chr19	38500000	42400000	0.0225892	40504605	40504770
