# Supplementary material for: Genomic Clustering of differential DNA methylated regions (epimutations) associated with the epigenetic transgenerational inheritance of disease and phenotypic variation
Source: BMC Genomics. 2016 Jun 1;17:418. doi: 10.1186/s12864-016-2748-5 (PMC4888261; doi:10.1186/s12864-016-2748-5)
Supplement: Additional file 5: Table S4. — Granulosa DMR clusters. DMR clusters with start-end, statistical significance for each DMR with start and stop information for the Granulosa Cell dataset. (PDF 35 kb) [file 12864_2016_2748_MOESM5_ESM.pdf]

Supplemental Table S4 **Granulosa Cell DMR Cluster**

Cluster Chromosome	ClusterStart	ClusterEnd	DMR min P Value	DMR cSTART	DMR cSTOP
chr1	78900000	82650000	0.017	79021827	79059686
chr1	78900000	82650000	0.0258	80663791	80682480
chr1	78900000	82650000	0.0013	80837906	80866672
chr1	78900000	82650000	0.0415	80867063	80876234
chr1	78900000	82650000	0.0023	82229581	82233249
chr1	85550000	87750000	0.0434	85771683	85773503
chr1	85550000	87750000	0.0408	85811563	85827563
chr1	85550000	87750000	0.0134	86076279	86085681
chr1	85550000	87750000	0.0063	87421576	87547908
chr1	94200000	97250000	0.0274	95284085	95288174
chr1	94200000	97250000	0.0415	95566712	95576151
chr1	94200000	97250000	0.0402	96038287	96055624
chr1	94200000	97250000	0.0277	96173539	96178485
chr1	161300000	164150000	0.0291	161584858	161620198
chr1	161300000	164150000	0.0275	162173279	162184423
chr1	161300000	164150000	0.0093	163212921	163214511
chr1	161300000	164150000	0.0179	163278970	163282815
chr1	161300000	164150000	0.0431	163319507	163327017
chr1	204850000	208800000	0.0198	205852746	205913024
chr1	204850000	208800000	0.0256	206438325	206455477
chr1	204850000	208800000	0.0489	206460327	206471241
chr1	204850000	208800000	0.0115	206815462	206817131
chr1	204850000	208800000	0.0326	207469265	207475013
chr1	204850000	208800000	0.0121	208489337	208509947
chr1	204850000	208800000	0.0037	208700770	208705093
chr1	210700000	213350000	0.0276	211391510	211391584
chr1	210700000	213350000	0.0363	211392892	211392958
chr1	210700000	213350000	0.0082	212489224	212506444
chr1	210700000	213350000	0.0074	212512692	212671238
chr1	210700000	213350000	0.0058	212670771	212685721
chr1	246800000	250050000	0.0143	247324252	247347553
chr1	246800000	250050000	0.011	248136869	248162256
chr1	246800000	250050000	0.0326	248727385	248733112
chr1	246800000	250050000	0.05	248764890	248775194
chr1	246800000	250050000	0.045	249358105	249371165
chr3	2800000	6450000	0.0459	3571500	3577157
chr3	2800000	6450000	0.0291	4390427	4419424
chr3	2800000	6450000	0.0485	4522915	4524733
chr3	2800000	6450000	0.0329	4760550	4769909
chr3	2800000	6450000	0.0026	4769882	4781027
chr3	2800000	6450000	0.0403	5808396	5808470
chr5	58000000	61150000	0.0097	58166155	58168558
chr5	58000000	61150000	0.0477	59162536	59165371
chr5	58000000	61150000	0.0397	59551937	59768901
chr5	58000000	61150000	0.0095	59972177	59972420
chr5	58000000	61150000	0.001	59994101	60003261

chr5	151350000	154850000	0.0224	152414554	152415938
chr5	151350000	154850000	0.0006	152877349	152878653
chr5	151350000	154850000	0.0184	153157638	153178179
chr5	151350000	154850000	0.0125	153298163	153308017
chr5	151350000	154850000	0.0465	154637426	154648795
chr5	170750000	174300000	0.0042	172393234	172421726
chr5	170750000	174300000	0.0158	172560734	172591208
chr5	170750000	174300000	0.0217	172684455	172685851
chr5	170750000	174300000	0.0082	172723907	172727994
chr7	112700000	116000000	0.0154	114077733	114100794
chr7	112700000	116000000	0.0005	114264017	114277780
chr7	112700000	116000000	0.0483	114500155	114524030
chr7	112700000	116000000	0.0076	114670493	114674335
chr10	13700000	16100000	0.0155	14131309	14134994
chr10	13700000	16100000	0.0432	15118871	15119001
chr10	13700000	16100000	0.0267	15384697	15397270
chr10	13700000	16100000	0.0283	15653948	15666851
chr10	32250000	36050000	0.0098	34067718	34067784
chr10	32250000	36050000	0.0336	34069763	34069830
chr10	32250000	36050000	0.0299	34132512	34140450
chr10	32250000	36050000	0.0083	34197630	34205252
chr10	62500000	65550000	0.0041	63564454	63571168
chr10	62500000	65550000	0.0249	63818778	63906406
chr10	62500000	65550000	0.0125	64292136	64307281
chr10	62500000	65550000	0.0061	64452384	64452452
chr10	86850000	90800000	0.042	86972428	87027794
chr10	86850000	90800000	0.0075	87849158	87868697
chr10	86850000	90800000	0.0139	88749392	88766001
chr10	86850000	90800000	0.003	88817241	88817795
chr10	86850000	90800000	0.046	89652342	89675785
chr10	86850000	90800000	0.0317	89716624	89743137
chr10	86850000	90800000	0.0108	90483845	90491463
chr10	103400000	107350000	0.0377	104553284	104596044
chr10	103400000	107350000	0.0417	105237637	105254750
chr10	103400000	107350000	0.0418	105342683	105351381
chr10	103400000	107350000	0.015	105357786	105389938
chr10	103400000	107350000	0.0349	105424839	105445294
chr10	103400000	107350000	0.0039	106249534	106273060
chr10	103400000	107350000	0.023	106280444	106304660
chr10	103400000	107350000	0.0382	106639912	106645579
chr15	31800000	34250000	0.0278	32312308	32325152
chr15	31800000	34250000	0.017	32527394	32533554
chr15	31800000	34250000	0.0207	32627631	32637861
chr15	31800000	34250000	0.05	33761485	33771148
chr17	6700000	8700000	0.0414	6781899	6845097
chr17	6700000	8700000	0.0141	7969379	7972877
chr17	6700000	8700000	0.0328	8037982	8039776
chr17	6700000	8700000	0.0448	8672024	8675258
chr20	2850000	6800000	0.027	4095745	4105509

chr20	2850000	6800000	0.03	4786264	4789596
chr20	2850000	6800000	0.0169	4790363	4800997
chr20	2850000	6800000	0.0091	4829535	4836708
chr20	2850000	6800000	0.0117	4843458	4846844
chr20	2850000	6800000	0.0433	4998627	5060283
chr20	2850000	6800000	0.0009	5089611	5097383
chr20	11650000	15150000	0.0309	12309334	12338494
chr20	11650000	15150000	0.0292	13221692	13225367
chr20	11650000	15150000	0.0167	13591783	13594256
chr20	11650000	15150000	0.0332	13596710	13621456
chr20	11650000	15150000	0.008	13599148	13603193
chrX	132950000	136400000	0.0496	134387285	134396762
chrX	132950000	136400000	0.0064	134474700	134502013
chrX	132950000	136400000	0.0009	134537438	134573364
chrX	132950000	136400000	0.0082	134914058	134925182
chrX	132950000	136400000	0.0438	134937891	134995705
