# Supplementary material for: Genomic Clustering of differential DNA methylated regions (epimutations) associated with the epigenetic transgenerational inheritance of disease and phenotypic variation
Source: BMC Genomics. 2016 Jun 1;17:418. doi: 10.1186/s12864-016-2748-5 (PMC4888261; doi:10.1186/s12864-016-2748-5)
Supplement: Additional file 6: Table S5. — Primordial germ cell DMR clusters. DMR clusters with start-end, statistical significance for each DMR with start and stop information regarding the Primordial Germ cell dataset. (PDF 36 kb) [file 12864_2016_2748_MOESM6_ESM.pdf]

Supplemental Table S5 **Primordial Germ Cell DMR Clusters**

Cluster Chromosome	ClusterStart	ClusterEnd	DMR min P Value	DMR cSTART	DMR cSTOP
chr1	159050000	164750000	0.036802	160171951	160172893
chr1	159050000	164750000	0.00792557	160208359	160209309
chr1	159050000	164750000	0.0273203	160580940	160581884
chr1	159050000	164750000	0.0311665	160626353	160627315
chr1	159050000	164750000	0.0436967	161007450	161008388
chr1	159050000	164750000	0.0213911	161040276	161041226
chr1	159050000	164750000	0.0217852	161229983	161230930
chr1	159050000	164750000	0.0304003	161340241	161341182
chr1	159050000	164750000	0.00809794	161347591	161348535
chr1	159050000	164750000	0.0294102	161825890	161826855
chr1	159050000	164750000	0.0143487	162116977	162117430
chr1	159050000	164750000	0.00393428	162590020	162590976
chr1	159050000	164750000	0.0279007	162626124	162627080
chr1	159050000	164750000	0.0184928	162772333	162773271
chr1	159050000	164750000	0.0251045	162857313	162858260
chr1	159050000	164750000	0.0217125	163907346	163908294
chr1	159050000	164750000	0.0124801	164521184	164532002
chr1	159050000	164750000	0.00888314	164521184	164532002
chr1	200550000	204200000	0.0194718	201390131	201404487
chr1	200550000	204200000	0.0197359	202257225	202257896
chr1	200550000	204200000	0.0429305	202328074	202369623
chr1	200550000	204200000	0.00372789	202368685	202435004
chr1	200550000	204200000	0.000921743	202510208	202530499
chr1	200550000	204200000	0.0406367	202540093	202540740
chr3	14800000	18250000	0.0289346	15686892	15687821
chr3	14800000	18250000	0.030706	16279260	16280198
chr3	14800000	18250000	0.0187567	16592988	16593980
chr3	14800000	18250000	0.00454576	16610435	16611391
chr3	14800000	18250000	0.0150627	16788100	16789041
chr3	14800000	18250000	0.0304174	16846045	16847028
chr3	70500000	74700000	0.0289723	70936978	70970216
chr3	70500000	74700000	0.00634754	71196037	71196963
chr3	70500000	74700000	0.0449891	71371559	71372491
chr3	70500000	74700000	0.0219333	71731367	71732845
chr3	70500000	74700000	0.0444849	72473788	72474717
chr3	70500000	74700000	0.0239694	72530654	72532116
chr3	70500000	74700000	0.0481186	72746147	72747171
chr3	70500000	74700000	0.00800012	73509299	73510216
chr3	70500000	74700000	0.0169808	74436160	74437236
chr3	70500000	74700000	0.0349111	74533124	74534053
chr3	70500000	74700000	0.000574555	74542152	74543081
chr3	96850000	99100000	0.0215823	96999191	97000108
chr3	96850000	99100000	0.00165115	97151439	97152377
chr3	96850000	99100000	0.0226279	97734478	97735416

chr3	96850000	99100000	0.0383649	98330456	98475753
chr3	96850000	99100000	0.00522096	98476626	98813129
chr3	96850000	99100000	0.00534158	98868908	98871060
chr4	68100000	71200000	0.0328797	68183927	68491288
chr4	68100000	71200000	0.0135619	69130593	69133655
chr4	68100000	71200000	0.0255994	69273932	69274038
chr4	68100000	71200000	0.0368125	69273932	69274038
chr4	68100000	71200000	0.000785821	70052319	70081449
chr4	68100000	71200000	0.0089517	70215672	70216598
chr4	68100000	71200000	0.0334207	70272395	70273333
chr4	68100000	71200000	0.013404	71072318	71077823
chr5	137300000	141200000	0.0113062	138425829	138426662
chr5	137300000	141200000	0.042222	139153024	139153962
chr5	137300000	141200000	0.0438338	139263023	139264762
chr5	137300000	141200000	0.0399221	139263023	139264762
chr5	137300000	141200000	0.00807352	139277596	139298139
chr5	137300000	141200000	0.0100801	139347062	139348071
chr5	137300000	141200000	0.00332865	139380269	139381842
chr5	137300000	141200000	0.0150889	139428888	139436813
chr6	137800000	141300000	0.015595	137890464	137892084
chr6	137800000	141300000	0.00722435	138438824	138439239
chr6	137800000	141300000	0.00415868	139361659	139362149
chr6	137800000	141300000	0.0323883	139527553	139528065
chr6	137800000	141300000	0.0308233	139764413	139765553
chr6	137800000	141300000	0.0183117	139773176	139773676
chr6	137800000	141300000	0.0462539	139805361	139992607
chr7	22150000	24500000	0.0166337	22583395	22667014
chr7	22150000	24500000	0.0131091	23085569	23086894
chr7	22150000	24500000	0.0140976	23085569	23086894
chr7	22150000	24500000	0.0329937	23409480	23463877
chr7	22150000	24500000	0.0100341	24145578	24147420
chr8	16200000	19200000	0.0349496	17207231	17208169
chr8	16200000	19200000	0.0242392	17279596	17280525
chr8	16200000	19200000	0.0266768	17367760	17368694
chr8	16200000	19200000	0.0124856	17446743	17447681
chr8	16200000	19200000	0.0239299	18151985	18153084
chr8	16200000	19200000	0.0469366	19105018	19108061
chr10	13150000	15600000	0.00633833	13620435	13620551
chr10	13150000	15600000	0.00637096	13725912	13727278
chr10	13150000	15600000	0.0118524	13971093	13972962
chr10	13150000	15600000	0.0416435	14740991	14747069
chr10	13150000	15600000	0.013841	15129367	15134072
chr10	33150000	36250000	0.0499723	34251973	34252914
chr10	33150000	36250000	0.029364	34279874	34280854
chr10	33150000	36250000	0.0173162	34740402	34741337
chr10	33150000	36250000	0.00607209	34832721	34833656
chr10	33150000	36250000	0.0488985	35139783	35140047

chr10	42950000	46550000	0.0184041	44085301	44093595
chr10	42950000	46550000	0.0299522	44397069	44398016
chr10	42950000	46550000	0.0149771	44590716	44591633
chr10	42950000	46550000	0.0120616	44716641	44717561
chr10	42950000	46550000	0.00301	44911284	44912204
chr10	42950000	46550000	0.0286629	44997581	44998510
chr10	42950000	46550000	0.0443392	46139837	46163246
chr10	86600000	90650000	0.0414848	86856617	86912521
chr10	86600000	90650000	0.0417371	88497766	88498065
chr10	86600000	90650000	0.0417371	88504916	88505215
chr10	86600000	90650000	0.0493252	88557017	88557409
chr10	86600000	90650000	0.0493252	88569311	88569703
chr10	86600000	90650000	0.0493252	88575672	88576064
chr10	86600000	90650000	0.0300812	88591541	88613240
chr10	86600000	90650000	0.00555123	88749392	88766001
chr10	86600000	90650000	0.0453932	88787902	88788468
chr10	86600000	90650000	0.0305101	88860843	88861391
chr10	86600000	90650000	0.0185495	89071915	89075732
chr10	86600000	90650000	0.00164727	90493477	90497050
chr15	25100000	32750000	0.00359595	26020147	26021052
chr15	25100000	32750000	0.00598212	26032642	26033583
chr15	25100000	32750000	0.0403975	26242461	26244145
chr15	25100000	32750000	0.0025765	26419130	26420059
chr15	25100000	32750000	0.0252395	27073756	27090445
chr15	25100000	32750000	0.00618495	28328085	28328568
chr15	25100000	32750000	0.0337836	28342453	28342962
chr15	25100000	32750000	0.0224698	28952117	28952709
chr15	25100000	32750000	0.0187091	29014486	29053344
chr15	25100000	32750000	0.0474358	29066728	29067352
chr15	25100000	32750000	0.0495567	29127688	29144513
chr15	25100000	32750000	0.0337836	29270459	29270968
chr15	25100000	32750000	0.0105507	29381627	29401328
chr15	25100000	32750000	0.0114594	29416742	29417241
chr15	25100000	32750000	0.00884557	29420195	29420696
chr15	25100000	32750000	0.0445708	29427627	29442041
chr15	25100000	32750000	0.00288001	29504338	29504925
chr15	25100000	32750000	0.00507221	29529870	29546854
chr15	25100000	32750000	0.0165708	29942062	29942571
chr15	25100000	32750000	0.00199533	29949860	29950385
chr15	25100000	32750000	0.0318432	30177249	30177765
chr15	25100000	32750000	0.0136383	30235979	30236780
chr15	25100000	32750000	0.0228597	30609336	30699773
chr15	25100000	32750000	0.015911	30806851	30807347
chr15	25100000	32750000	0.0199121	31124736	31125235
chr15	25100000	32750000	0.0410577	31142307	31142826
chr15	25100000	32750000	0.0185007	31179580	31180129
chr15	25100000	32750000	0.0299075	32334774	32336256
