# Supplementary material for: Genomic Clustering of differential DNA methylated regions (epimutations) associated with the epigenetic transgenerational inheritance of disease and phenotypic variation
Source: BMC Genomics. 2016 Jun 1;17:418. doi: 10.1186/s12864-016-2748-5 (PMC4888261; doi:10.1186/s12864-016-2748-5)
Supplement: Additional file 7: Table S6. — Sperm and DMR clusters. DMR clusters with start-end, statistical significance for each DMR with start and stop information regarding clusters for the sperm DMR dataset. (PDF 41 kb) [file 12864_2016_2748_MOESM7_ESM.pdf]

Supplemental Table S6 **Sperm DMR Clusters**

Cluster Chromosome	ClusterStart	ClusterEnd	DMR min P Value	DMR cSTART	DMR cSTOP
chr1	76500000	81650000	0.00000633	76716621	76717399
chr1	76500000	81650000	3.21E-08	77218978	77219578
chr1	76500000	81650000	6.32E-16	77218978	77219578
chr1	76500000	81650000	0.00000382	78344996	78345976
chr1	76500000	81650000	1.33E-16	78450272	78451687
chr1	76500000	81650000	1.26E-19	78450272	78451687
chr1	76500000	81650000	1.33E-16	78450272	78451687
chr1	76500000	81650000	1.26E-19	78450272	78451687
chr1	76500000	81650000	5.16E-09	78450592	78451687
chr1	76500000	81650000	0.000000028	79005645	79006565
chr1	76500000	81650000	0.0000002	79058741	79060044
chr1	76500000	81650000	0.00000623	79680291	79680891
chr1	76500000	81650000	0.00000426	79910185	79910863
chr1	76500000	81650000	0.00000337	79910185	79910863
chr1	76500000	81650000	0.000000337	80274216	80275308
chr1	76500000	81650000	3.63E-10	80557664	80558744
chr1	76500000	81650000	0.00000801	80834920	80835615
chr1	92800000	97400000	0.000000004	93660805	93661590
chr1	92800000	97400000	0.000000404	93734203	93734913
chr1	92800000	97400000	0.000000113	93784998	93785998
chr1	92800000	97400000	0.00000151	93866197	93866982
chr1	92800000	97400000	0.00000195	94792412	94793122
chr1	92800000	97400000	0.00000195	94792412	94793122
chr1	92800000	97400000	0.00000971	94866084	94867099
chr1	92800000	97400000	0.0000036	94866199	94867099
chr1	92800000	97400000	0.000000019	95403348	95404143
chr1	92800000	97400000	4.09E-08	95488479	95489374
chr1	92800000	97400000	0.00000256	95892653	95894255
chr1	92800000	97400000	0.00000371	96038195	96038795
chr1	92800000	97400000	0.00000793	96264926	96265526
chr1	92800000	97400000	0.00000832	96527328	96528133
chr1	250450000	253300000	0.00000704	250472051	250472651
chr1	250450000	253300000	1.79E-11	251260561	251261161
chr1	250450000	253300000	1.46E-19	251356410	251357495
chr1	250450000	253300000	2.07E-27	251356410	251357495
chr1	250450000	253300000	9.89E-09	252412922	252413522
chr1	250450000	253300000	0.00000209	252412922	252413522
chr1	250450000	253300000	9.89E-09	252412922	252413522
chr1	250450000	253300000	0.00000209	252412922	252413522
chr1	250450000	253300000	0.00000188	252646829	252647729
chr2	180500000	183550000	0.00000155	181416132	181417032

chr2	180500000	183550000	0.000000909	181615695	181616678
chr2	180500000	183550000	0.000000909	181615695	181616678
chr2	180500000	183550000	2.42E-10	181715756	181716441
chr2	180500000	183550000	0.00000111	181715841	181716441
chr2	180500000	183550000	2.98E-11	182460590	182461570
chr2	180500000	183550000	1.04E-12	182460590	182461570
chr4	160550000	162600000	0.00000242	160665157	160666372
chr4	160550000	162600000	7.46E-09	160931316	160932317
chr4	160550000	162600000	2.16E-10	160931316	160932317
chr4	160550000	162600000	0.00000623	161285353	161286243
chr4	160550000	162600000	1.08E-11	162504664	162505639
chr4	160550000	162600000	8.81E-09	162504664	162505639
chr5	170750000	174650000	0.00000557	171103703	171104382
chr5	170750000	174650000	0.00000696	172630371	172631649
chr5	170750000	174650000	0.000000118	172749107	172749987
chr5	170750000	174650000	0.00000133	172749107	172749787
chr5	170750000	174650000	0.000000118	172749107	172749987
chr5	170750000	174650000	0.00000133	172749107	172749787
chr5	170750000	174650000	0.000000601	172951361	172952061
chr5	170750000	174650000	0.00000376	173066362	173067163
chr6	106850000	110750000	0.00000107	107547373	107547973
chr6	106850000	110750000	0.00000418	108168967	108169567
chr6	106850000	110750000	0.00000479	108515786	108516671
chr6	106850000	110750000	2.08E-29	108814406	108815606
chr6	106850000	110750000	2.08E-29	108814406	108815606
chr6	106850000	110750000	0.00000155	108814526	108815306
chr6	106850000	110750000	9.14E-25	108814526	108815606
chr6	106850000	110750000	0.00000155	108814526	108815306
chr6	106850000	110750000	9.14E-25	108814526	108815606
chr6	106850000	110750000	0.000000705	109793195	109793870
chr7	150000	4000000	0.00000172	2006927	2007527
chr7	150000	4000000	0.00000172	2006927	2007527
chr7	150000	4000000	0.00000347	2104216	2104816
chr7	150000	4000000	0.00000347	2104216	2104816
chr7	150000	4000000	0.000000386	2229865	2231454
chr7	150000	4000000	0.0000058	2230364	2231254
chr7	150000	4000000	0.00000427	21141850	21142450
chr7	150000	4000000	0.00000342	21141850	21142570
chr7	150000	4000000	0.00000638	28112035	28112635
chr7	150000	4000000	0.000000176	31001102	31001702
chr7	113000000	115850000	0.0000001	11424819	11425640
chr7	113000000	115850000	9.99E-12	11424819	11425640
chr7	113000000	115850000	0.0000001	11424819	11425640
chr7	113000000	115850000	9.99E-12	11424819	11425640
chr7	113000000	115850000	8.44E-09	11583824	11584614

chr7	113000000	115850000	2.72E-10	11584014	11584614
chr7	113000000	115850000	4.94E-09	11584014	11584614
chr7	113000000	115850000	1.14E-09	114277254	114278461
chr7	113000000	115850000	0.00000181	114502670	114504375
chr7	113000000	115850000	1.89E-13	114503595	114504375
chr7	113000000	115850000	0.000000763	114953948	114955044
chr7	113000000	115850000	3.57E-10	114953948	114955044
chr7	126200000	129400000	0.00000168	127442049	127442649
chr7	126200000	129400000	8.60E-13	127449653	127450253
chr7	126200000	129400000	0.00000248	127772817	127773697
chr7	126200000	129400000	0.000000362	127816023	127816717
chr7	126200000	129400000	3.72E-18	127816023	127816717
chr7	126200000	129400000	0.000000209	128173824	128174714
chr7	140800000	143150000	1.39E-08	141214859	141215936
chr7	140800000	143150000	3.05E-08	141470621	141471436
chr7	140800000	143150000	0.000000135	142249435	142250435
chr7	140800000	143150000	0.00000185	142276341	142277241
chr7	140800000	143150000	7.51E-08	142789367	142789967
chr7	140800000	143150000	3.02E-11	142790359	142790959
chr8	60400000	62600000	0.000000209	60673757	60674357
chr8	60400000	62600000	3.31E-09	60673757	60674357
chr8	60400000	62600000	1.87E-09	60673757	60674357
chr8	60400000	62600000	0.000000189	61607737	61608550
chr8	60400000	62600000	5.55E-09	62361422	62362307
chr8	60400000	62600000	1.66E-08	62361422	62362307
chr10	54700000	58650000	0.00000711	55855898	55856498
chr10	54700000	58650000	0.00000553	56132674	56133469
chr10	54700000	58650000	6.92E-10	56204571	56206172
chr10	54700000	58650000	0.00000616	56378095	56379587
chr10	54700000	58650000	0.000000329	56675163	56676245
chr10	54700000	58650000	1.63E-10	56678345	56679370
chr10	54700000	58650000	0.00000223	56678445	56679248
chr10	54700000	58650000	5.05E-08	56687180	56687875
chr10	54700000	58650000	0.00000309	56720709	56721596
chr10	54700000	58650000	0.00000067	57077243	57078332
chr10	54700000	58650000	0.00000067	57077243	57078332
chr10	54700000	58650000	0.000008	57548762	57549583
chr10	54700000	58650000	4.80E-10	57549874	57551068
chr10	83050000	87500000	3.78E-08	83476529	83477129
chr10	83050000	87500000	0.000000267	84021838	84023150
chr10	83050000	87500000	0.00000337	84714200	84715211
chr10	83050000	87500000	3.95E-08	84714285	84715211
chr10	83050000	87500000	0.00000565	85016559	85017435
chr10	83050000	87500000	2.16E-16	85032294	85033304
chr10	83050000	87500000	8.25E-17	85032294	85033194

chr10	83050000	87500000	3.37E-08	85032294	85033194
chr10	83050000	87500000	1.09E-08	85078411	85079388
chr10	83050000	87500000	1.08E-08	85586351	85587341
chr10	83050000	87500000	3.37E-08	85586351	85587341
chr10	83050000	87500000	0.00000226	86320851	86321529
chr10	83050000	87500000	0.00000195	86320851	86321529
chr10	83050000	87500000	0.00000234	86323498	86324403
chr10	83050000	87500000	0.00000281	86551715	86552513
chr11	80500000	84350000	0.00000441	82426660	82427380
chr11	80500000	84350000	0.00000205	82429579	82430269
chr11	80500000	84350000	4.28E-08	82429579	82430269
chr11	80500000	84350000	5.58E-11	82470316	82471209
chr11	80500000	84350000	0.00000972	82470413	82471209
chr11	80500000	84350000	2.71E-08	82470413	82471209
chr11	80500000	84350000	0.00000013	82519950	82520632
chr12	14850000	18750000	6.01E-17	16815952	16816847
chr12	14850000	18750000	2.69E-13	16815952	16816847
chr12	14850000	18750000	0.00000125	16824203	16825214
chr12	14850000	18750000	0.00000125	16824203	16825214
chr12	14850000	18750000	0.00000076	16824308	16825214
chr12	14850000	18750000	0.00000076	16824308	16825214
chr12	14850000	18750000	0.00000955	16829845	16831365
chr15	25200000	28250000	3.16E-23	26056221	26058016
chr15	25200000	28250000	1.29E-10	26336470	26337157
chr15	25200000	28250000	5.65E-14	26337866	26338466
chr15	25200000	28250000	2.44E-10	26395012	26396497
chr15	25200000	28250000	1.92E-12	27105574	27106174
chr15	25200000	28250000	2.21E-11	27184787	27185387
chr15	25200000	28250000	1.27E-23	27346566	27347666
chr15	25200000	28250000	5.23E-20	27346660	27347666
chr20	1000000	6200000	3.30E-17	2776113	2776813
chr20	1000000	6200000	0.00000181	2776113	2776813
chr20	1000000	6200000	1.94E-20	2984086	2984883
chr20	1000000	6200000	1.94E-20	2984086	2984883
chr20	1000000	6200000	1.91E-13	2984169	2984883
chr20	1000000	6200000	1.91E-13	2984169	2984883
chr20	1000000	6200000	0.00000238	2987593	2988193
chr20	1000000	6200000	0.00000238	2987593	2988193
chr20	1000000	6200000	9.48E-15	3416588	3417188
chr20	1000000	6200000	2.79E-12	3416588	3417188
chr20	1000000	6200000	7.68E-21	3442365	3443350
chr20	1000000	6200000	0.00000208	3442365	3443350
chr20	1000000	6200000	0.00000447	3497439	3498621
chr20	1000000	6200000	0.00000155	3497439	3498621
chr20	1000000	6200000	2.07E-30	3580516	3581116

chr20	1000000	6200000	0.00000375	3667367	3668263
chr20	1000000	6200000	0.00000253	4047954	4048958
chr20	1000000	6200000	0.000000376	4103885	4104485
chr20	1000000	6200000	1.90E-12	4220107	4221198
chr20	1000000	6200000	0.00000158	4259416	4260219
chr20	1000000	6200000	6.66E-08	4789651	4790543
chr20	1000000	6200000	6.66E-08	4789651	4790543
chr20	1000000	6200000	0.000000148	5022398	5022998
chr20	1000000	6200000	0.00000858	5179159	5179759
chr20	1000000	6200000	0.0000068	6067533	6068428
chr20	1000000	6200000	0.00000192	28130915	28131715
chr20	1000000	6200000	0.0000021	29546601	29547322
chrUn	23350000	27100000	0.00000205	25163917	25164597
chrUn	23350000	27100000	3.17E-14	25163917	25164597
chrUn	23350000	27100000	1.40E-10	25163917	25164597
chrUn	23350000	27100000	3.62E-08	25163917	25164597
chrUn	23350000	27100000	6.03E-10	25300105	25300805
chrUn	23350000	27100000	7.39E-10	25300105	25300805
